# An Automated Decision Support System for Portfolio Allocation Based on Mutual Information and Financial Criteria

**DOI:** 10.3390/e27050480

**Published:** 2025-04-29

**Authors:** Massimiliano Kaucic, Renato Pelessoni, Filippo Piccotto

**Affiliations:** 1 Department of Economic, Business, Mathematical and Statistical Sciences, University of Trieste, 34127 Trieste, Italy; renato.pelessoni@deams.units.it (R.P.); filippo.piccotto@phd.units.it (F.P.); 2 SOFI Lab, Soft Computing Laboratory for Finance and Insurance, University of Trieste, 34127 Trieste, Italy

**Keywords:** mutual information, portfolio optimization, risk budgeting, DISH-XX, knowledge-based financial management system

## Abstract

This paper introduces a two-phase decision support system based on information theory and financial practices to assist investors in solving cardinality-constrained portfolio optimization problems. Firstly, the approach employs a stock-picking procedure based on an interactive multi-criteria decision-making method (the so-called TODIM method). More precisely, the best-performing assets from the investable universe are identified using three financial criteria. The first criterion is based on mutual information, and it is employed to capture the microstructure of the stock market. The second one is the momentum, and the third is the upside-to-downside beta ratio. To calculate the preference weights used in the chosen multi-criteria decision-making procedure, two methods are compared, namely equal and entropy weighting. In the second stage, this work considers a portfolio optimization model where the objective function is a modified version of the Sharpe ratio, consistent with the choices of a rational agent even when faced with negative risk premiums. Additionally, the portfolio design incorporates a set of bound, budget, and cardinality constraints, together with a set of risk budgeting restrictions. To solve the resulting non-smooth programming problem with non-convex constraints, this paper proposes a variant of the distance-based parameter adaptation for success-history-based differential evolution with double crossover (DISH-XX) algorithm equipped with a hybrid constraint-handling approach. Numerical experiments on the US and European stock markets over the past ten years are conducted, and the results show that the flexibility of the proposed portfolio model allows the better control of losses, particularly during market downturns, thereby providing superior or at least comparable ex post performance with respect to several benchmark investment strategies.

## 1. Introduction

The portfolio selection process typically involves two stages. The first phase comprises the selection of the most promising stocks to be included in the optimization, while the second concerns the optimal wealth allocation between the portfolio constituents.

Ranking and selecting stocks from an investment basket is a challenge that has been addressed in the literature in several ways, such as traditional stock-picking techniques based on factor models [1,2] or novel approaches based on machine learning techniques [3,4]. When the stock-picking process involves many different and conflicting financial criteria, it can fall into the realm of multi-criteria decision-making (MCDM) problems. This ensemble of methods remarkably supports the portfolio selection practice since it provides a comprehensive range of techniques that tackle issues related to the stock-picking phase. In recent times, various multi-criteria decision-making methods have been employed to rank superior securities and construct optimal portfolios, such as the preference ranking organization method for enrichment evaluation [5,6], the technique for order of preference by similarity to ideal solution [7,8], and the multi-criteria optimization and compromise solution [9], to mention some of the most often used. For a comparison of the performance of several multi-criteria decision-making techniques, we refer the reader to [10,11]. To account for the decision-makers’ irrational exuberance when making decisions in the presence of risk and uncertainty, ref. [12] initiated the TODIM (the Portuguese acronym for interactive multi-criteria decision-making) method. Specifically, this approach incorporates prospect theory’s principles [13] to define the value function that ranks between criteria alternatives, considering investors’ behavioral characteristics. Recently, the TODIM method has gained increasing interest in the literature. In [14], the authors applied this procedure to rank 462 equities from the constituents of the S&P500 Index by adopting nine financial criteria. They considered several portfolio cardinalities and constructed equally weighted or ranking-based weighted portfolios, finding that investments built through this technique yielded better results in terms of the Sharpe ratio. Ref. [15] extended this framework by embedding the TODIM method in a multi-objective portfolio selection based on the mean–variance paradigm to optimize the portfolio constituents, finding promising results. Their proposed model has been tested on a limited number of assets from the Chinese stock market.

Although still highly influential, Markowitz’s pioneering mean–variance framework [16] faces numerous practical challenges when applied in real-world scenarios. Moreover, the need to meet the demands of practitioners and institutional investors, who are increasingly engaged in the portfolio design process, has resulted in considerable research aimed at developing alternative optimization approaches. In particular, the so-called risk parity framework has become a mainstream asset allocation approach, gaining widespread relevance in both industry and academia [17,18]. This strategy allocates wealth in such a way that the risk contribution per asset to the portfolio risk is equalized, focusing on managing the different sources of risk involved in the investment process and introducing the idea of risk diversification. However, the existence and uniqueness of a solution to the risk parity portfolio problem is guaranteed only in some particular cases [19,20]. Furthermore, optimizing a portfolio to reach risk parity compliance neglects the performance dimension of an investment, which represents a necessity for most categories of investors. Consequently, some authors have proposed asset allocation problems with different performance objectives while imposing the parity condition as a portfolio constraint [21,22]. It is worth noting that risk parity is a special case of the more general risk budgeting approach, where assets are given a predetermined weight, called a budget, as a percentage of the total portfolio risk [23,24].

**Figure 1:**
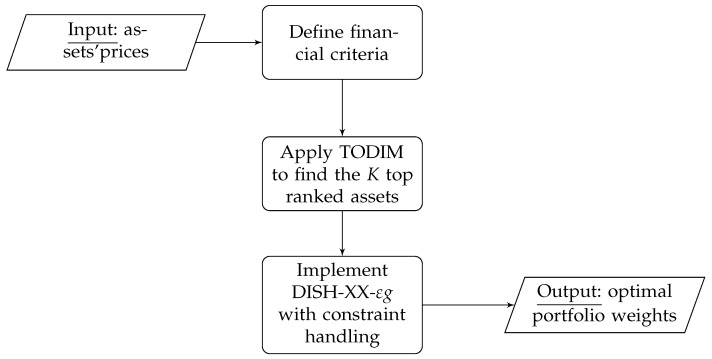
Structure of the knowledge-based financial management system.

This paper presents a novel automated decision support system built upon two interconnecting modules tailored to solving portfolio optimization models that maximize a financial performance measure subject to real-world constraints, particularly cardinality constraints. This knowledge-based structure is illustrated in Figure 1. The first module employs the TODIM procedure to develop a ranking between stocks concerning several financial criteria identified by the end user. With this step, we can bypass the explicit management of the cardinality constraint within the optimization process. In the second module, we determine the optimal portfolio weights using a version of the linear population size reduction success-based differential evolution algorithm with double crossover (DISH-XX [25]). The synergy between these two modules can address the necessities of various types of end users who wish to be directly involved in the design of the portfolio strategy. The system developed in this paper parallels the one proposed in [26], with the differences being (i) a more practical approach aimed at supporting practitioners, (ii) its application in a single- and not a multi-objective setting, and (iii) the consideration of information theory—and, in particular, entropy—in choosing the portfolio constituents.

Furthermore, the capabilities of our automated financial management system are evaluated by examining two instances of a portfolio optimization problem, where the objective function to optimize is a modified version of the Sharpe ratio [27]. In this refined performance measure, when the portfolio excess return is negative, it is multiplied by the standard deviation instead of being divided. This version aligns with the risk–return preferences of a rational investor, even if the risk premiums are negative [28]. We consider the outlook of an institutional investor or a portfolio manager who seeks to operate in large equity markets by selecting a limited pool of stocks to form a portfolio with a suitable performance while maintaining risk diversification. To achieve this goal, we introduce the following real-world constraints. Firstly, a cardinality constraint limits the number of assets to a reasonable portfolio size. Then, a budget constraint ensures that we allocate all of the available capital, while box constraints prescribe lower and upper bounds on the fraction of capital invested in each asset. The resulting portfolio model is similar to the one analyzed in [29], where the authors empirically proved notable ex post financial profitability regarding many ex post performance metrics. In the second instance, along with the previous restrictions, this work introduces the direct control of the portfolio risk in the optimization phase using a set of risk budgeting constraints. More precisely, a tolerance threshold allowing for minor upper and lower deviations from the parity is adopted, resulting in a formulation similar to the one developed in [30]. This approach relaxes the risk parity conditions and provides greater flexibility in determining the risk contributions of the portfolio constituents. The results are mixed-integer optimization problems that belong to the family of cardinality-constrained portfolio optimization problems, which are NP-hard [31]. In addressing the challenge posed by the resolution of this particular class of optimization problems, a number of researchers in the fields of finance and computer science have directed their attention towards metaheuristics, given their demonstrated simplicity and effectiveness [32]. For instance, the beetle antennae search algorithm has attracted significant interest due to its computational efficiency and global convergence properties, rendering it suitable for solving portfolio optimization problems subject to real-world constraints such as transaction costs and cardinality limits [33]. Furthermore, the integration of artificial neural dynamics into portfolio optimization models has demonstrated substantial enhancements in computational efficiency and solution accuracy when compared to traditional methodologies [34,35]. The present work falls within this line of research, focusing on the differential evolution algorithm (DE [36]), a metaheuristic extensively used to solve single- and multi-objective asset allocation problems [37,38]. Specifically, it considers a recently developed augmented version of DE, called distance-based parameter adaptation for success-history-based differential evolution with double crossover (DISH-XX), which has shown very competitive results based on several benchmark functions from real-world engineering problems [25]. Regarding the current literature and knowledge, this paper is the first to apply this algorithm to portfolio optimization. Section 2.1 provides a detailed description of the progression from DE to DISH-XX. Since the original version of this solver is blind to non-bound constraints, it is equipped with an ensemble of constraint-handling techniques. Specifically, a repair mechanism is used to manage box and budget constraints, as in [39]. Subsequently, for risk budgeting constraints, the proposed constraint-handling approach accelerates the convergence process towards the feasible region by applying a gradient-based mutation [40] and uses the ε-constrained method [41] to transform the constrained optimization problem into an equivalent unconstrained one.

Note that selecting the alternative criteria for the preliminary stock-picking phase and establishing their relative preferences constitutes a crucial phase for the TODIM method. Specifically, this work considers a complementary set of three financial criteria, each providing a unique perspective. Firstly, it adopts a peripherality measure for the stocks based on mutual information, similar to the approach discussed in [42]. The mutual information dimension focuses on the microstructure of the stock market to capture the full spectrum of assets’ dependencies. Recently, many scholars have investigated the capabilities of mutual information and entropy as investors’ tools to make choices under uncertain conditions. Section 2.2 provides an overview of the most recent literature contributions in this field. Then, the second and third criteria are the momentum based on the most recent monthly returns and the upside-to-downside beta ratio, respectively. The former highlights the ability of stocks to generate value over time, and the latter assesses the responsiveness of a stock concerning market upswings and downswings. Furthermore, to calculate the preference weights associated with the three aforementioned criteria, this work exploits two approaches. The first consists of a static method where the three criteria have the same importance, without introducing any relative preference. Next, the second technique uses the joint entropic information carried by criteria and evaluates their contributions, dynamically adjusting the relative preferences during the investment period.

The following are the main literature contributions of this paper.

- We develop a knowledge-based financial management system to solve cardinality-constrained portfolio optimization problems. This expert system is built upon two interconnected modules. On the one hand, a multi-criteria decision analysis technique called TODIM handles the cardinality constraint. On the other hand, the DISH-XX algorithm is extended with an ensemble of constraint-handling techniques and a gradient-based mutation.
- This study introduces two portfolio selection models where the objective function to maximize is a modified version of the Sharpe ratio under some real-world constraints. The first instance considers cardinality, box, and budget constraints. The second one introduces a set of risk budgeting constraints to provide explicit control of risk.
- When running the TODIM procedure for the preliminary ranking, we use three complementary financial criteria, namely the peripherality measure based on mutual information, the momentum measure, and the upside-to-downside beta ratio.
- To set up the relative preference weights of the three criteria, an equally weighted method and an entropy-based method are adopted.
- An extensive experimental analysis is conducted considering the two most significant indices of the American and European stock markets, namely the S&P 500 and the STOXX Europe 600.
- The empirical part validates the profitability of our investment strategy considering several ex post performance metrics and compares the two portfolio models described above against some alternatives that pre-select the stocks using the criteria individually, as well as the market benchmark.

The remainder of this paper is structured as follows. Section 2 presents some related works regarding the differential evolution algorithm and the use of information theory for portfolio optimization. Section 3 illustrates the two instances of the portfolio model. Section 4 and Section 5 describe the two modules that compose the developed decision support system. Section 6 presents the experimental analysis, discussing the data and investment setup and showing the ex post performance results. Finally, Section 7 concludes the paper, summarizing the main findings, illustrating potential research limitations, and suggesting some future research directions.

## 2. Related Works

### 2.1. From DE to DISH-XX

The differential evolution algorithm works according to the following steps. The algorithm starts by randomly sampling an initial population of candidate solutions. Then, it iteratively produces new trial vectors through mutation and crossover phases. If a new individual outperforms the original one, it survives and progresses to the next generation. This iterative process continues until it satisfies some stopping conditions, and the algorithm returns the best-found solution to the optimization problem. The original algorithm, developed in [36], includes three user-defined control parameters: the population size NP, the scaling factor *F*, and the crossover rate Cr. Over recent decades, scholars have proposed several enhanced versions of this algorithm. These advancements typically involve using refined mutation schemes, introducing external archives to store the most promising solutions, and adaptively determining the parameters NP, *F*, and Cr. For a comprehensive survey of the latest advancements in the field of differential evolution-based algorithms, see [43]. In [44], the authors introduced an influential variant that uses a control parameter adaptation strategy. Specifically, this version samples the parameters *F* and Cr from a probability distribution and stores successful values in an external archive. An improved version of the latter, called success-history-based differential evolution, was proposed in [45]. Instead of sampling *F* and Cr from gradually adapted probability distributions, the authors proposed to use historical archives ($M_{F}$ and $M_{Cr}$) to store effective parameter values from recent generations. The algorithm then generates new *F* and Cr parameters by sampling near these stored pairs. Next, the same authors introduced a linearly decreasing function that adaptively reduces the population size over the generations [46]. In [47], the authors proposed an update to the scaling factor and a crossover rate adaptation that exploits information from the Euclidean distance between the trial and the original individual. They called this new algorithm distance-based success history differential evolution (DISH), proving its superior performance over several versions of DE. The DISH-XX algorithm [25] used as a solver in this paper is a refined version of DISH that introduces a secondary crossover between the trial vector and one of the historically best-found solutions randomly selected from the archive.

### 2.2. Information Theory in Portfolio Optimization

Metrics from information theory, especially entropy, have led to significant literature contributions in portfolio theory. For instance, several authors have used entropy as a proxy for portfolio risk, starting with the seminal paper [48]. To mention some recent works, ref. [49] proposed a return–entropy portfolio model and compared it with the classical Markowitz strategy. The work in [50] prescribes a setup for portfolio optimization where entropy and mutual information are used instead of variance and covariance as risk measurements. In this approach, the mutual information measures the statistical independence between two random variables, and it is used as a more general approach to capture nonlinear relationships [51]. Along with using entropy as a risk measure, several contributions have used this metric to quantify portfolio diversification. In [52], the authors proposed a model that aims to maximize the entropy of the portfolio weight vector, extending the classical Markowitz framework by adding a control on diversification. In [53], this approach has been broadened by suggesting a mean–variance–skewness–entropy multi-objective optimization model. More recently, in [54], the authors managed the mean, variance, and entropy objectives using a self-adapting parameter λ that adjusts to the market conditions.

In recent times, researchers in finance have considered markets as networks in which stocks correspond to nodes and the links are related to the correlations of returns. In [55], the authors used network theory to select stocks from the peripheral regions of the financial filtered networks, finding that they performed better than stocks belonging to the networks’ central zones. The work in [56] bridges the gap between the mean–variance and network theories. In particular, a negative relationship between optimal portfolio weights and the centrality of assets in the financial market network has been evidenced. In [57], the authors tested various dependence measures, such as the Pearson and Kendall correlations and lower tail dependence, to construct interconnected graphs and build optimal mean–variance portfolios. Moreover, a trend has emerged in the literature where, instead of using canonical correlation measures, researchers employ mutual information to capture nonlinear dependencies among stocks and describe the microstructure of the financial market. The foundational work in this area is the paper [58], where the authors constructed minimum spanning trees based on the mutual information between stocks in the Chinese stock market. By applying this methodology and combining it with the approach suggested in [55], some authors have considered a measure of asset centrality based on mutual information and have proposed various stock-picking techniques for portfolio construction [42]. This paper follows the latter approach to establish one of the three criteria employed within the multi-criteria decision-making module.

## 3. Portfolio Models

### 3.1. Investment Strategy Setup

This paper considers a frictionless market that does not allow for short selling, and all investors act as price takers. The investable universe consists of n≥2 risky assets, and a portfolio is denoted by the vector of weights $w={\left(w_{1},\ldots ,w_{n}\right)}^{⊤}\in R^{n}$, where $w_{i}$ represents the proportion of capital invested in asset *i*, with i=1,⋯,n. $R_{i}$ indicates the random variable representing the rate of return of asset *i*, and $\mu_{i}$ is its expected value. Hence, the random variable $R_{p}\left(w\right)=\sum_{i=1}^{n}w_{i}R_{i}$ expresses the portfolio rate of return, while the expected rate of return of portfolio w is defined as

(1) $$\mu_{p}\left(w\right)=\sum_{i=1}^{n}w_{i}\mu_{i}$$

and its volatility is given by

(2) $$\sigma_{p}\left(w\right)=\sqrt{\sum_{i=1}^{n}\sum_{j=1}^{n}\sigma_{ij}w_{i}w_{j}}=\sqrt{w^{⊤}\Sigma w}$$

where ${\left(\Sigma \right)}_{ij}=\sigma_{ij}$ is the covariance between assets *i* and *j*, with i,j=1,⋯,n, with the covariance matrix Σ assumed to be positive definite. Since investors perceive large deviations from the portfolio mean value as damaging, Equation (2) represents the so-called portfolio risk.

Given this framework, a portfolio that provides the maximum return for a given level of risk or, equivalently, has the minimum risk for a given level of return is called efficient. This decision-making approach is widely known as mean–variance analysis, and the set of optimal mean–variance trade-offs in the risk–return space forms the efficient frontier [16]. In this setting, the so-called Sharpe ratio identifies the best investment among efficient portfolios. This performance measure is defined as

(3) $$SR\left(w\right)=\frac{\mu_{p}\left(w\right)-r_{f}}{\sigma_{p}\left(w\right)}$$

and expresses the net compensation, with respect to a risk-free rate $r_{f}$, earned by the investor per unit of risk. However, the reliability of this performance measure decreases when the portfolio excess return $\mu_{p}\left(w\right)-r_{f}$ is negative, since (in some cases) an investor would select a higher-risk portfolio using the Sharpe ratio. To overcome this issue, the proposed portfolio selection model considers the so-called modified Sharpe ratio [28], defined as

(4) $$MSR\left(w\right)=\frac{\mu_{p}\left(w\right)-r_{f}}{\sigma_{p}{\left(w\right)}^{\mathrm{sign}(\mu_{p}\left(w\right)-r_{f})}}$$

where sign(z) is the sign function of z∈R. Observe that, if the portfolio excess return is non-negative, the modified Sharpe ratio is equal to the Sharpe ratio. Otherwise, it multiplies the portfolio excess return by the standard deviation. In this manner, even in periods of market downturn, portfolios with lower risk and a higher excess return will be preferred.

### 3.2. First Proposed Model

This paper considers a portfolio model that is similar to the one inspected in [29]. The aim is to maximize the modified Sharpe ratio illustrated in Equation (4) subject to the following real-world constraints.

- *Budget*. Since all available capital needs to be invested at each investment window, the following holds:
  (5) $$\sum_{i=1}^{n}w_{i}=1.$$
- *Cardinality*. The portfolio includes exactly *K* assets, where K≤n. To model the inclusion or exclusion of the *i*th asset in the portfolio, a binary variable $\delta_{i}$ is introduced as
  $$\delta_{i}=\left\{\begin{array}{c} 0,\;\mathrm{if}\;\mathrm{asset}\;i\;\mathrm{is}\;\mathrm{excluded}\\ 1,\;\mathrm{if}\;\mathrm{asset}\;i\;\mathrm{is}\;\mathrm{included} \end{array}\right.$$
  for i=1,…,n, where $\delta ={\left(\delta_{1},\ldots ,\delta_{n}\right)}^{⊤}\in {\left\{0,1\right\}}^{n}$, and the cardinality constraint can be written as
  (6) $$\sum_{i=1}^{n}\delta_{i}=K.$$
  Then, $I_{K}=\left\{i\in \left\{1,\cdots ,n\right\}:w_{i}>0\right\}$ denotes the set of active portfolio weights, with $|I_{K}|=K$.
- *Box*. A balanced portfolio should avoid extreme positions and foster diversification. Hence, maximum and minimum limits for portfolio weights are imposed, expressed by
  (7) $$\delta_{i}\;lb_{i}\leq w_{i}\leq \delta_{i}\;ub_{i},\;i=1,\ldots ,n\;,$$
  where $l_{i}$ and $u_{i}$ are the lower and upper bounds for the weight of the *i*th asset, respectively, with $0<lb_{i}<ub_{i}\leq 1$ to exclude short sales.

The resulting is a mixed-integer optimization model that requires some ad hoc techniques to be practically handled. In this paper, instead of directly handling the cardinality constraint in the optimization process, as in [29,31], the TODIM procedure described in Section 4 is used to perform preliminary stock selection and bypass the cardinality issue. Then, a metaheuristic is employed to search for optimal solutions for the reduced portfolio allocation problem.

### 3.3. Risk Budgeting Approach

To control the degree of risk-based diversification between the portfolio constituents, the risk budgeting portfolio [23] is introduced. This approach allocates the risk according to the profile described by the vector $b={\left(b_{1},\cdots ,b_{n}\right)}^{⊤}$, with $0<b_{i}<1$, i=1,…,n, and $\sum_{i=1}^{n}b_{i}=1$, such that

(8) $$RC_{i}\left(w\right)=b_{i}\sigma_{p}\left(w\right)\;\forall i\;,$$

where $RC_{i}\left(w\right)=\frac{w_{i}{\left(\Sigma w\right)}_{i}}{\sqrt{w^{⊤}\Sigma w}}$ denotes the risk contribution of the *i*-th stock to the portfolio risk.

Notice that the risk budgeting approach represents a relaxation of the more restrictive risk parity conditions, since the risk parity portfolio occurs when $b_{i}=1\;/\;n$ for all *i*. Appendix A.1 illustrates in detail the basics of the risk parity framework.

### 3.4. Proposed Risk Budgeting Formulation for the Second Portfolio Model

Inspired by the risk budgeting setup, this paper designs an investment strategy in which the deviations from risk parity are fixed by the investor’s risk profile according to the following set of inequalities:

(9) $$\left(1-\underline{\nu }\right)\frac{\sigma_{p}\left(w\right)}{n}\leq RC_{i}\left(w\right)\leq \left(1+\bar{\nu }\right)\frac{\sigma_{p}\left(w\right)}{n}\;\forall i$$

where $0\leq \underline{\nu }\leq \bar{\nu }<1$. Note that, for $\underline{\nu }=\bar{\nu }=0$, Equation (9) reduces to the risk parity condition, while increasing values of these parameters introduce portfolios with increasing deviations of the risk contributions from the parity condition and thus greater risk concentrations. It can be proven that any optimization problem that involves the use of Equation (9) is non-convex. More details regarding the non-convexity of the risk budgeting formulation are given in Appendix A.2.

Summing up, the second instance of the portfolio model that optimizes the MSR measure of Equation (4) considers budget, bound, and cardinality constraints as specified in Equations (5)–(7), while introducing the direct control of the portfolio risk according to the set of constraints outlined in Equation (9).

## 4. Multi-Criteria Decision Analysis Module

To address the cardinality constraint (6) and develop a portfolio model involving only real variables, the proposed approach selects assets with higher rankings based on a set of criteria, using the TODIM method.

### 4.1. TODIM Generalities

The TODIM method facilitates decision-making by evaluating the importance of each criterion according to the subjective preferences of each investor. It consists of the following sequential steps.

1. *Constructing the multi-criteria decision-making matrix between criteria and alternatives.* Given *m* alternatives $\left\{a_{1},\cdots ,a_{m}\right\}$ and *s* criteria $\left\{c_{1},\cdots ,c_{s}\right\}$, the decision matrix $A={\left(a_{i,j}\right)}_{m\times s}$ is expressed as
  $$A=\left(\begin{array}{cccc} a_{1,1} & a_{1,2} & \cdots & a_{1,s}\\ a_{2,1} & a_{2,2} & \cdots & a_{2,s}\\ \vdots & \vdots & \ddots & \vdots \\ a_{m,1} & a_{m,2} & \cdots & a_{m,s} \end{array}\right)$$
  where $a_{i,j}$ is the performance evaluation of $a_{i}$ under criterion $c_{j}$.
2. *Determining the criteria weights*. In this step, the criteria weighting vector $\beta ={\left(\beta_{1},\cdots ,\beta_{s}\right)}^{⊤}$, which satisfies $0\leq \beta_{j}\leq 1$ and $\sum_{j}\beta_{j}=1$, needs to be determined. This vector defines the relative preference degree of the procedure toward the *s* criteria. Two weighting schemes are analyzed in this paper. The first assigns the same weight to each criterion to avoid any prior preference for a specific criterion in the TODIM structure. The second one utilizes the entropy weight method [59]. The contribution of the alternative $a_{i}$ to the criterion $c_{j}$ is calculated as
  (10) $$\lambda_{i,j}=\frac{a_{i,j}}{\sum_{i=1}^{m}a_{i,j}}\;,\;i=1,\ldots ,m\;\mathrm{and}\;j=1,\ldots ,s.$$
  Next, the entropy value $en_{j}$ for the *j*th criterion is given by
  (11) $$en_{j}=-\frac{1}{\ln(m)}\sum_{i=1}^{m}\lambda_{i,j}\ln\left(\lambda_{i,j}\right)\;,\;j=1,\ldots ,s$$
  where $en_{j}$ denotes the total contribution of all alternatives to criterion $c_{j}$. If $\lambda_{i,j}=0$, it follows that $\lambda_{i,j}\ln\left(\lambda_{i,j}\right)=0$. After obtaining the entropy values, the entropy weight $\beta_{j}$ is
  (12) $$\beta_{j}=\frac{1-en_{j}}{s-\sum_{j=1}^{s}en_{j}}\;,\;j=1,\ldots ,s.$$
3. *Binning and normalizing criteria matrix*. The third step transforms the raw criteria matrix *A* into a different matrix, $A^{\prime }$, by binning each element into 10 bins. Specifically, if a criterion is considered a benefit, a value of 10 is assigned to the alternatives in the top 10% for that criterion. Conversely, if the criterion is a cost, a value of 10 is assigned to the alternatives in the bottom 10%. Then, to make the scores comparable, a normalization procedure is used to obtain the normalized values $N_{i,j}^{\prime }$.
4. *Computing alternative comparisons*. Through the normalized scores, the alternatives can be compared based on their overall scores across the criteria. For criterion $c_{j}$, the criteria score of alternative $a_{i}$ against alternative $a_{k}$ is defined as in [60]
  (13) $$CS_{j}\left(a_{i},a_{k}\right)=\left\{\begin{array}{cc} \beta_{j}{\left(N_{i,j}^{\prime }-N_{k,j}^{\prime }\right)}^{\eta_{1}} & \mathrm{if}\;N_{i,j}^{\prime }\geq N_{k,j}^{\prime }\\ -\xi \beta_{j}{\left(N_{k,j}^{\prime }-N_{i,j}^{\prime }\right)}^{\eta_{2}} & \mathrm{if}\;N_{i,j}^{\prime }<N_{k,j}^{\prime } \end{array}\right.$$
  where $\beta_{j}$ is the objective weight of criterion $c_{j}$; $\eta_{1},\eta_{2}\in \left[0,1\right]$ are the two risk parameters of the value function in the domain of gains and losses; and ξ>0 is the loss aversion coefficient in the loss domain.
  After calculating the dominance degree with respect to criterion $c_{j}$ between any two alternatives $a_{i}$ and $a_{j}$ using Equation (13), the final comparison score concerning each criterion is
  (14) $$FS_{j}\left(a_{i}\right)=\sum_{k=1}^{m}CS_{j}\left(a_{i},a_{k}\right)\;\;i=1,\ldots ,m\;\mathrm{and}\;j=1,\ldots ,s.$$
5. *Determining the final ranking between alternatives.* In the last step, the rank of each alternative $a_{i}$ is obtained as
  (15) $$R\left(a_{i}\right)=\sum_{j=1}^{s}FS_{j}\left(a_{i}\right).$$
  The procedure then concludes with the normalization of the final ranks. These range between 0 and 1, with the most preferred alternative having a value of 1 and the least preferred having a value of 0.

### 4.2. Application of TODIM to Investable Universe

The previously described multi-criteria decision-making method is applied to the assets that are part of the investable universe, representing the alternatives $a_{i}$, using three criteria based on the financial performance of stocks, which will be described in the experimental section. Given this procedure, the cardinality constraint can be tackled by picking the *K* assets with higher rankings, and, with this procedure, we can express the portfolio optimization problem without the auxiliary vector δ. To avoid any confusion, the notation of this paper uses a vector $x\in R^{K}$ of *K* components instead of w to express weights, and we write the two inspected models as follows:

(16) $$\begin{array}{cc} \underset{x\in R^{K}}{\mathrm{maximize}}\; & f(x)=MSR(x)\\ s.t.\; & \sum_{i=1}^{K}x_{i}=1\\ \; & lb_{i}\leq x_{i}\leq ub_{i},\;i=1,\ldots ,K \end{array}$$

and, for the risk budgeting model,

(17) $$\begin{array}{cc} \underset{x\in R^{K}}{\mathrm{maximize}}\; & f(x)=MSR(x)\\ s.t.\; & \sum_{i=1}^{K}x_{i}=1\\ \; & lb_{i}\leq x_{i}\leq ub_{i},\;i=1,\ldots ,K\\ \; & g_{j}\left(x\right)\leq 0,\;j=1,\ldots ,2K \end{array}$$

where $g_{j}\left(x\right)=x^{⊤}\left(\frac{\left(1-\underline{\nu }\right)}{K}\Sigma^{\left(K\right)}-E_{j}^{\left(K\right)}\right)x$ for j=1,…,K and $g_{j}\left(x\right)=x^{⊤}\left(E_{j-K}^{\left(K\right)}-\frac{\left(1+\bar{\nu }\right)}{K}\Sigma^{\left(K\right)}\right)x$ for j=K+1,…,2K, with $\Sigma^{\left(K\right)}\in R^{K\times K}$ being the covariance matrix of the *K* assets selected by TODIM, $E_{j}^{\left(K\right)}=\frac{1}{2}\left(e_{i}^{\left(K\right)}e_{i}^{{\left(K\right)}^{⊤}}\Sigma^{\left(K\right)}+\Sigma^{\left(K\right)}e_{i}^{\left(K\right)}e_{i}^{{\left(K\right)}^{⊤}}\right)$, and $e_{i}^{\left(K\right)}\in R^{K}$ denotes the *j*th column of the identity matrix.

## 5. Optimization Module

This section introduces the developed version of the DISH-XX algorithm specifically designed to solve Problems (16) and (17).

### 5.1. DISH-XX Algorithm

The following steps outline the core components of the DISH-XX algorithm as presented in [25] for the problem of optimizing a generic function $f:R^{K}\to R$.

1. *Initialization.* At iteration t=0, the algorithm commences with the initialization of a random population P consisting of $NP_{init}$ solutions. During this step, additional parameters are configured: the final population size ($NP_{f}$), the maximum number of objective function evaluations ($FES_{max}$), and two parameters utilized in the mutation operator ($\alpha_{max}$ and $\alpha_{min}$). Moreover, two external archives are introduced: the first, denoted as *A*, stores solutions that have been improved by the corresponding trial vectors; the second, $A_{best}$, contains the most promising solutions. Based on the prescriptions given in [25], two historical memory arrays of size *H*, $M_{F}$ and $M_{Cr}$, are defined component-wise as
  (18) $$M_{F,h}=0.5\;\mathrm{for}\;h=1,\ldots ,H-1,\;\mathrm{and}\;M_{F,H}=0.9$$
  and
  (19) $$M_{Cr,h}=0.8\;\mathrm{for}\;h=1,\ldots ,H-1,\;\mathrm{and}\;M_{Cr,H}=0.9$$
  which will be used to define the values for the scaling factor *F* and the crossover rate Cr.
2. *Mutation.* For each generation t≥0, the mutation operator used in DISH-XX is the current-to-pbest-*w*/1 strategy. Let $FES_{ratio}$ be the ratio between the current number of objective function evaluations FES and $FES_{max}$. The mutation vector $v^{p}$ for each individual *p* is then generated as follows:
  (20) $$v^{p}=x^{p}+F_{w}^{p}\;\left(x^{pbest}-x^{p}\right)+F^{p}\;\left(x^{r_{1}}-x^{r_{2}}\right)$$
  where $x^{pbest}$ is one of the 100α% best solutions in the archive $A_{best}$, with $\alpha =FES_{ratio}\;\left(\alpha_{max}-\alpha_{min}\right)+\alpha_{min}$; $x^{r_{1}}$ is randomly selected from the current population P and $x^{r_{2}}$ from P∪A. It is worth noting that $i\neq pbest\neq r_{1}\neq r_{2}$. The scaling factor $F^{p}$ is generated from a Cauchy distribution with location parameter $M_{F,\tilde{r}}$ randomly selected from the historical memory array $M_{F}$ and a scale parameter value of 0.1. If the generated value $F^{p}$ is non-positive, it is drawn again, and, if it is greater than 1, it is set to 1. In addition, to bound its value in the exploration phase, we set $F^{p}=0.7$ whenever $FES_{ratio}<0.6$ and $F^{p}>0.7$. The weighted scaling factor $F_{w}^{p}$ depends on $F^{p}$ and $FES_{max}$ as follows:
  $$F_{w,i}=\left\{\begin{array}{cc} 0.7\;F^{p}, & \mathrm{if}\;FES_{ratio}<0.2\\ 0.8\;F^{p}, & \mathrm{if}\;FES_{ratio}<0.4\\ 1.2\;F^{p}, & \mathrm{otherwise}. \end{array}\right.$$
  This mutation strategy combines a greedy approach in the first difference and an exploratory factor in the second difference.
3. *Double Crossover.* The DISH-XX algorithm employs a double crossover mechanism. The first crossover is the standard binomial crossover as in [36], which combines the mutation vector $v^{p}$ with the target vector $x^{p}$ to produce a temporary trial vector $u^{*,p}={\left(u_{1}^{*,p},\ldots ,u_{K}^{*,p}\right)}^{⊤}$. This process is based on the crossover rate value $Cr^{p}$, which is randomly generated using a normal distribution with a mean value $M_{Cr,\tilde{\tilde{r}}}$, randomly selected from the memory array $M_{Cr}$, and a standard deviation value of 0.1. The $Cr^{p}$ value is then bounded between 0 and 1, with values outside this range truncated to the nearest bound. Similarly to the scaling factor, the crossover rate depends on $FES_{ratio}$ as follows:
  $$Cr^{p}=\left\{\begin{array}{cc} \max\{Cr^{p},\;0.7\}, & \mathrm{if}\;FES_{ratio}<0.25\\ \max\{Cr^{p},\;0.6\}, & \mathrm{if}\;FES_{ratio}<0.5\\ Cr^{p}, & \mathrm{otherwise}. \end{array}\right.$$
  The second crossover involves the archive of historically best-found solutions $A_{best}$, enhancing the diversity and exploration capabilities of the algorithm. Using the same value $Cr^{p}$ of the first crossover, the trial vector $u^{p}={\left(u_{1}^{p},\ldots ,u_{K}^{p}\right)}^{⊤}$ is generated component-wise as follows:
  (21) $$u_{i}^{p}=\left\{\begin{array}{cc} u_{i}^{*,p} & \mathrm{if}\;rand_{j}\leq Cr^{p}\;\mathrm{or}\;j=j_{rand}\\ x_{i}^{rbest} & \mathrm{otherwise} \end{array}\right.$$
  where $rand_{j}$ is a uniformly distributed random number, $j_{rand}$ is a randomly chosen index in {1,…,K}, and $x^{rbest}={\left(x_{1}^{rbest},\ldots ,x_{K}^{rbest}\right)}^{⊤}$ is a solution randomly selected from $A_{best}$.
4. *Selection.* The selection process in DISH-XX is based on the comparison of the trial vector $u^{p}$ and the target vector $x^{p}$. The objective function values of both vectors are evaluated, and the one with the better fitness value is selected for the next generation. This ensures that the population evolves toward better solutions over time.
5. *Adaptation of Control Parameters.* DISH-XX incorporates adaptive mechanisms for control parameters, such as the scaling factor and the crossover rate. These parameters are adjusted based on the success history of previous generations, allowing the algorithm to dynamically adapt to the problem landscape and enhance its performance. After each generation, one cell in both memory arrays is updated. DISH-XX uses an index *k* to track which cell will be updated. The index is initialized to 1, so, after the first generation, the first memory cell is updated. The index is incremented by one after each update, and, when it exceeds the value of *H*, it resets to 1. There is one exception to this update process: the last cell in both arrays is never updated and retains a value of 0.9 for both control parameters. Let $S_{F}$ and $S_{Cr}$ be arrays storing successful $F^{p}$ and $Cr^{p}$, respectively. A pair $\left(F^{p},Cr^{p}\right)$ is considered successful if it generates a trial vector $u^{p}$ that outperforms the target vector $x^{p}$. The size of $S_{F}$ and $S_{Cr}$ is a random number between 0 (indicating that no trial vector is better than the target) and NP (indicating that all trial vectors are better than their targets). Consequently, the value stored in the *k*th cell of the memory arrays after a given generation is
  (22) $$M_{F,k}=\left\{\begin{array}{cc} {\mathrm{mean}}_{WL}\left(S_{F}\right) & \mathrm{if}\;S_{F}\neq \emptyset \;\mathrm{and}\;k\neq H\\ M_{F,k} & \mathrm{otherwise} \end{array}\right.$$
  and
  (23) $$M_{Cr,k}=\left\{\begin{array}{cc} {\mathrm{mean}}_{WL}\left(S_{Cr}\right) & \mathrm{if}\;S_{Cr}\neq \emptyset \;\mathrm{and}\;k\neq H\\ M_{Cr,k} & \mathrm{otherwise} \end{array}\right.$$
  where ${\mathrm{mean}}_{WL}$ is the weighted Lehmer mean of the corresponding control parameter $S_{F}$ and $S_{Cr}$ and is defined as
  $${\mathrm{mean}}_{WL}\left(S\right)=\frac{\sum_{n=1}^{\left|S\right|}\omega_{n}\;S_{n}^{2}}{\sum_{n=1}^{\left|S\right|}\omega_{n}\;S_{n}}$$
  for $S\in \{S_{F},S_{Cr}\}$. The weights $\omega_{n}$ are computed as the Euclidean distance between the trial vector $u^{p}$ and the the individual $x^{p}$; specifically,
  (24) $$\omega_{n}=\frac{\sqrt{\sum_{i=1}^{K}{\left(u_{i}^{n}-x_{i}^{n}\right)}^{2}}}{\sum_{m=1}^{\left|S\right|}\sqrt{\sum_{i=1}^{K}{\left(u_{i}^{m}-x_{i}^{m}\right)}^{2}}}.$$
  This weighting scheme encourages exploitation while aiming to prevent the premature convergence of the algorithm to local optima.
6. *Decrease in the Population Size.* The population size dynamically reduces during the execution of the algorithm to allocate more time for exploration in the later stages of optimization. Specifically, at the end of each generation, the population size is updated using the following formula:
  (25) $$NP=NP_{int}-FES_{ratio}\;\left(NP_{init}-NP_{f}\right).$$
7. *Population and Archive Management.* The archive of historically best-found solutions $A_{best}$ is maintained throughout the optimization process. The archive is periodically updated with the best solutions available, ensuring that it remains relevant and effective. The population P and the archive *A* adjust their sizes in response to changes in (25) by removing the worst-ranking individuals.
8. *Termination.* The algorithm iterates through the above steps until a termination criterion is met. Common termination criteria include reaching a maximum number of generations, achieving a satisfactory fitness level, or observing no significant improvement over a predefined number of iterations.

### 5.2. Dealing with Budget and Box Constraints

In the construction phase of portfolio models, admissible solutions have to satisfy budget and buy-in threshold constraints. However, DISH-XX is blind to these constraints. To overcome this issue, the algorithm is equipped with a hybrid constraint-handling procedure. At first, to guarantee feasibility with respect to the bound constraints (7), this study introduces the following random combination [61]:

(26) $$x_{i}^{p}=\left\{\begin{array}{cc} \left(1-r\right)\;ub_{i}+r\;x_{i}^{p}, & \mathrm{if}\;x_{i}^{p}>ub_{i}\\ \left(1-r\right)\;lb_{i}+r\;x_{i}^{p}, & \mathrm{if}\;x_{i}^{p}<lb_{i} \end{array}\right.$$

where p=1,⋯,NP and i=1,⋯,K. Then, it uses the repair transformations developed in [39] to also satisfy the budget constraint (5). The assumptions needed to apply this method are the following:

1. $lb_{i}\leq x_{i}^{p}\leq ub_{i}$    ∀i;
2. $\sum_{i=1}^{K}lb_{i}<1$;
3. $\sum_{i=1}^{K}ub_{i}>1$.

Then, for each p=1,⋯,NP, the candidate solution $x^{p}$ is adjusted component-wise

(27) $$x_{i}^{p}=\left\{\begin{array}{c} l_{i}+\frac{\left(x_{i}^{p}-lb_{i}\right)}{\sum_{j=1}^{K}\left(x_{j}^{p}-lb_{i}\right)}\left(1-\sum_{j=1}^{K}lb_{i}\right),\;\mathrm{if}\;\sum_{j=1}^{K}x_{j}^{p}>1\\ x_{i}^{p},\;\mathrm{if}\;\sum_{j=1}^{K}x_{j}^{p}=1\;\\ u_{i}-\frac{\left(ub_{i}-x_{i}^{p}\right)}{\sum_{j=1}^{K}\left(ub_{i}-x_{j}^{p}\right)}\left(\sum_{j=1}^{K}ub_{i}-1\right),\;\mathrm{if}\;\sum_{j=1}^{K}x_{j}^{p}<1 \end{array}\right.$$

for all i∈1,⋯,K. As proven in [39], solutions transformed through Equation (27) fulfill, at the same time, budget and box constraints.

### 5.3. Dealing with Risk Budgeting Constraints

Dealing with the risk budgeting constraints in Problem (17) requires the definition of a proper constraint violation function. Given a candidate solution x, this quantity is defined as

(28) $$\phi \left(x\right)=\sum_{j=1}^{2K}\Delta g_{j}\left(x\right)$$

where $\Delta g_{j}\left(x\right)=\max\left\{0,g_{j}\left(x\right)\right\}$ represents the constraint violation for the *j*th inequality constraint, with j=1,…,2K.

Then, the ε-constrained method proposed in [41] transforms the constrained optimization model into an unconstrained one. More specifically, let $x^{1},x^{2}\in R^{K}$ be two candidate solutions with objective function values $f\left(x^{1}\right),f\left(x^{2}\right)$ and constraint violations $\phi (x^{1})$ and $\phi (x^{2})$, respectively. Then, the ε-comparison of the two solutions is defined as

(29) $$\left(f\left(x^{1}\right),\phi \left(x^{1}\right)\right){<}_{\varepsilon }\left(f\left(x^{2}\right),\phi \left(x^{2}\right)\right)\Leftrightarrow \left\{\begin{array}{cc} f\left(x^{1}\right)<f\left(x^{2}\right) & if\phi \left(x^{1}\right),\phi \left(x^{2}\right)\leq \varepsilon ,\\ f\left(x^{1}\right)<f\left(x^{2}\right) & if\phi \left(x^{1}\right)=\phi \left(x^{2}\right),\\ \phi \left(x^{1}\right)<\phi \left(x^{2}\right) & \mathrm{otherwise} \end{array}\right.$$

and

(30) $$\left(f\left(x^{1}\right),\phi \left(x^{1}\right)\right)\leq_{\varepsilon }\left(f\left(x^{2}\right),\phi \left(x^{2}\right)\right)\Leftrightarrow \left\{\begin{array}{cc} f\left(x^{1}\right)\leq f\left(x^{2}\right) & if\phi \left(x^{1}\right),\phi \left(x^{2}\right)\leq \varepsilon ,\\ f\left(x^{1}\right)\leq f\left(x^{2}\right) & if\phi \left(x^{1}\right)=\phi \left(x^{2}\right),\\ \phi \left(x^{1}\right)<\phi \left(x^{2}\right) & \mathrm{otherwise}. \end{array}\right.$$

It is worth noting that if both compared solutions are feasible or slightly infeasible (as determined by the ε value in the first parts of Equations (29) and (30)), or even if they have the same sum of constraint violation, they are compared using the values of the objective function. Conversely, if both solutions are infeasible, they are compared using the sum of their constraint violations. It is interesting to see that, if ε=∞, the ε-level comparison works by using as comparison criterion only the objective function values. If ε=0, then the ε-level comparison is equivalent to a lexicographic ordering in which the minimization of the sum of the constraint violation precedes the minimization of the objective function.

#### 5.3.1. Controlling the ε-Level

This study uses the following scheme based on  [41,62] to control the ε parameter:

(31) $$\begin{array}{cc} \varepsilon (0) & =\mathrm{mean}\left(\phi (P_{\mathrm{best}\;\mathrm{half}})\right)\\ \varepsilon (t) & =\left\{\begin{array}{cc} \varepsilon \left(0\right){\left(1-\frac{t}{T_{c}}\right)}^{cp} & \;0<t<T_{c},\\ 0 & \;t\geq T_{c}, \end{array}\right. \end{array}$$

where the initial ε-level is equal to the mean constraint violation of the best half of the initial population, $\mathrm{mean}\left(\phi (P_{\mathrm{best}\;\mathrm{half}})\right)$. The level is then updated until the iteration counter *t* reaches a maximum $T_{c}$. After this, the ε-level is set to 0. To maintain the stability and efficiency of the algorithm, cp is set equal to 5 and $T_{c}$ is given by

$$T_{c}=\left\{\begin{array}{cc} 0.2\;T_{max}, & \mathrm{if}\;N_{int}=18\;K\\ \frac{6\;FES_{max}}{K^{\sqrt{5}}}, & \mathrm{otherwise} \end{array}\right.$$

with $T_{max}$ representing the maximum number of iterations corresponding to $FES_{max}$.

#### 5.3.2. Gradient-Based Mutation

The gradient-based mutation is an operator that was first developed in [41], following the seminal work presented in [40]. The main idea of the method is to utilize the gradient information of the constraints to repair the infeasible candidate solutions, moving them toward the feasible region.

Given a candidate solution x, the vector of the values of the inequality constraint functions is $C\left(x\right)={\left(g_{1}\left(x\right),...,g_{2K}\left(x\right)\right)}^{⊤}$, and $\Delta C\left(x\right)={\left(\Delta g_{1}\left(x\right),...,\Delta g_{2K}\left(x\right)\right)}^{⊤}$ denotes the vector of the constraint violations. Next, the aim is to solve the following system of linear equations:

∇C(x)Δx=−ΔC(x)

where the values of the increments Δx are the variables and ∇C(x) is the gradient matrix of C(x),

$$\nabla C\left(x\right)=\left(\begin{array}{ccc} \frac{\partial g_{1}\left(x\right)}{\partial x_{1}} & \cdots & \frac{\partial g_{1}\left(x\right)}{\partial x_{K}}\\ \vdots & \ddots & \vdots \\ \frac{\partial g_{2K}\left(x\right)}{\partial x_{1}} & \cdots & \frac{\partial g_{2K}\left(x\right)}{\partial x_{K}} \end{array}\right)\;.$$

The Moore–Penrose pseudoinverse $\nabla C{\left(x\right)}^{+}$ gives an approximate solution as follows:

(32) $$\Delta x=-\nabla C{\left(x\right)}^{+}\Delta C\left(x\right)\;.$$

Thus, the new mutated solution can be written as

(33) $$x^{new}=x+\Delta x.$$

This repair operation is executed with a probability $P_{g}$ at every *K* iterations and is repeated for a maximum of $R_{g}$ times while the point is not feasible. In the numerical experiments, $P_{g}=0.2$ and $R_{g}=1$. Notice that only non-zero elements of ΔC(x) are repaired using this mutation.

### 5.4. The Proposed DISH-XX-εg Algorithm

In summary, the developed solver involves the following steps. When individuals are subject to mutation and crossover, the repair operator defined by Equations (26) and (27) manages budget and bound constraints (5) and (7) simultaneously. The risk budgeting inequality constraints, when incorporated into portfolio design, are addressed using the ε-constraint method, with the ε comparison (29) and the update rule (31) for the parameter ε. Then, the gradient-based mutation operator defined by Equations (32) and (33) leverages the gradient information of the risk budgeting constraints to accelerate the convergence of solutions toward the feasible region, as suggested in [40]. Finally, a pair $\left(F^{p},Cr^{p}\right)$ is considered successful if it generates a trial vector $u^{p}$ that outperforms the target vector $x^{p}$ in terms of the ε comparison (29). The same order relation is also used to update the population and the archives. The resulting enhanced DISH-XX algorithm with gradient-based mutation, denoted DISH-XX-εg, is illustrated in Appendix B in terms of addressing Problem (17). The termination criterion employed is the maximum number of objective function evaluations.

The parameter setup of the DISH-XX-εg algorithm is based on the recommendations of [25,62]. The maximum number of objective function evaluations depends on the problem dimension according to the following formula:

(34) $$FES_{max}=\left\{\begin{array}{cc} 1\times 10^{5}, & \mathrm{if}\;K\leq 10\\ 2\times 10^{5}, & \mathrm{if}\;10<K\leq 30\\ 4\times 10^{5}, & \mathrm{if}\;30<K\leq 50\\ 8\times 10^{5}, & \mathrm{if}\;50<K\leq 150\\ 1\times 10^{6}, & \mathrm{if}\;K>150. \end{array}\right.$$

Similarly, the initial population size is $N_{int}=50\;\ln\left(K\right)\sqrt{K}$ and the final population size is $N_{f}=4$. For the mutation parameters, $\alpha_{max}=0.25$ and $\alpha_{min}=\alpha_{max}/2=0.125$. The external archive *A* and the archive of the historical best solutions $A_{best}$ are initialized as empty. The historical memory size *H* is set to 5. Additionally, the termination criterion will be based on the maximum number of objective function evaluations. However, to avoid unnecessary computational costs in financial applications, the algorithm will terminate if the objective function value of the best solution, $x^{best}$, does not show a significant improvement over 10 consecutive iterations, indicating convergence.

## 6. Experimental Analysis

This section provides a detailed description of the numerical analysis aimed at evaluating the flexibility and effectiveness of the proposed automated expert system in managing the two instances of the modified Sharpe ratio-based portfolio model.

### 6.1. Data Set Description and Experimental Setup

The empirical analysis conducted in this work focuses on the American and European stock markets. Specifically, for the former, the daily closing prices of the constituents of the S&P 500 index for the period from 31 December 2014 to 31 October 2024 are considered. The latter case study refers to the securities listed in the STOXX Europe 600 index for the same period. Assets presenting missing data within the observation window have been discarded. As a result, the American dataset comprises 470 stocks, while the European investment basket includes 535 stocks.

The two case studies consider a rolling window investment plan with monthly portfolio rebalancing, with an out-of-sample window consisting of 94 months, covering the period from 31 January 2017 to 31 October 2024. For each month in this window, a historical approach based on the last two years of daily observations is adopted to calculate the expected rates of return and the covariance matrix. For each month of the investment phase, the DISH-XX-*ε*g solver is used to find the optimal wealth allocation in terms of portfolio weights. Appendix C illustrates the solving capabilities of the proposed algorithm. Table 1 recaps the data set structure and the experimental setup.

Regarding the portfolio designs, the risk-free rate of return $r_{f}$ in the objective function (4) is set to zero, as in [63], and the buy-in thresholds $lb_{i}$ and $ub_{i}$ are equal to 0.005 and 0.1, respectively. The cardinality parameter *K* is expressed as a fraction $K_{\%}$ of the number of assets in a given data set, i.e., $K=\lfloor K_{\%}\cdot n\rfloor$, and $K_{\%}$ is set equal to 5%, 10%, and 15%. Furthermore, in the risk budgeting model, $\nu =\bar{\nu }=\underline{\nu }$ in Equation (9), considering symmetrical ranges of deviation from the risk parity level. Three entries for the parameter ν are studied, namely 0.01, 0.05, and 0.10, where a higher value indicates more flexibility in the management of risk budgets. Note that this paper does not compare the introduced risk budgeting approach with the classical risk parity portfolio model; however, the choice of ν=0.01 represents a scenario with minimal deviations from the parity, which indirectly relates the results to it. Finally, regarding the practical implementation of the TODIM method, this study follows the suggestions in [60] by setting the parameters $\eta_{1}=\eta_{2}=0.88$ and the attenuation factor ξ=2.25 in Equation (13).

### 6.2. Criteria Used for the Screening of Assets

The preliminary stock-picking phase considers three complementary criteria to implement in the TODIM procedure. The first focuses on the microstructure of the stock market to capture the full spectrum of assets’ dependencies based on mutual information (MI). The so-called momentum measure (MOM) is the second criterion, which exploits the ability of individual stocks to generate value over time. The third metric consists of the upside-to-downside beta ratio (U/D ratio), which assesses the responsiveness of a stock with respect to upward and downward market movements. In the following, a description of the methodology employed to define and compute these three measures is provided.

#### 6.2.1. Eigenvector Centrality Measure Based on Mutual Information

The definition of the first criterion needs some preliminary notions about the Shannon entropy measure. Given a continuous random variable *X* with probability density function p(x), its entropy is defined as

$$H\left(X\right)=-\int_{x}p\left(x\right)\log p\left(x\right)\;dx.$$

Similarly, if one considers two continuous random variables *X* and *Y*, their joint entropy is given by

$$H\left(X,Y\right)=-\int_{x}\int_{y}p\left(x,y\right)\log p\left(x,y\right)\;dx\;dy$$

where p(x,y) is the joint probability density function of *X* and *Y*. The mutual information between two random variables captures the mutual dependence between them. For continuous variables, it is expressed as

$$MI\left(X,Y\right)=\int_{x}\int_{y}p\left(x,y\right)\log\frac{p(x,y)}{p(x)p(y)}\;dx\;dy$$

and it is zero if and only if they are independent.

Then, the dissimilarity between the rates of return of two stocks, namely $R_{j}$ and $R_{k}$, with j,k=1,…,n and j≠k, is quantified by the so-called normalized distance metric, defined as

(35) $$d\left(R_{j},R_{k}\right)=1-\frac{MI(R_{j},R_{k})}{H(R_{j},R_{k})}\;.$$

Notice that this distance ranges from 0 (perfect dependence) to 1 (independence), making it particularly useful in building networks. The last two years of daily observations are employed for the estimation of the distance $d(R_{j},R_{k})$, and, following the approach described in [42], we consider a minimum spanning tree (MST), defined as a connected subgraph that spans all nodes of a graph with the minimum total edge weight and no cycles. To construct the MST based on (35), Prim’s algorithm is used—a well-known method that, starting from an arbitrary node, iteratively connects it with the shortest edge until all nodes are included.

Once the MST is constructed, to identify key nodes within the network, we exploit eigenvector centrality, a measure that assigns an importance score to each node based on its connections. This centrality measure is obtained by computing the Perron eigenvector of the adjacency matrix of the MST, which corresponds to the principal eigenvalue. A high score of centrality characterizes influential stocks that are important nodes in their respective clusters, facilitating the transfer of information. However, because of their importance in the dynamics of the market, these stocks are more susceptible to market volatility. In contrast, nodes with low centrality scores are located on the periphery of the network, making them less susceptible to market risk and thus representing effective candidates for portfolio selection [55].

#### 6.2.2. Momentum Measure

Let $R_{i}$ be the random variable expressing the stochastic rate of return of stock *i* for a given period. The momentum of a stock is typically defined as the observed rate of return over a specified observation window that consists of *N* periods that begins in $t_{0}$ and ends in $t_{N}$:

(36) $$MOM_{i}\left(t_{1},t_{N}\right)=\prod_{t=t_{0}}^{t_{N-1}}\left(1+r_{i,t}\right)-1\;$$

where $r_{i,t_{1}},\cdots ,r_{i,t_{N}}$ are *N* consecutive observations of $R_{i}$. Stocks with higher momentum values are preferred. The momentum of a stock is calculated considering the last two years of monthly observations.

#### 6.2.3. Upside-to-Downside Beta Ratio

In financial analysis, the downside beta ($\beta^{-}$) measures the sensitivity of an asset to market returns when they are below a certain threshold. This beta component is particularly useful in evaluating the risk of an asset in adverse market conditions, and a percentage of the portfolio to stocks with low downside betas provides protection against market downturns [64]. Conversely, the upside beta ($\beta^{+}$) refers to periods when the market returns are higher than a threshold and reflects the potential gain capability of an asset during favorable market conditions. By considering this measure, investors can identify growth opportunities in order to construct portfolios that capitalize on market upswings. $R_{B}$ is a random variable that expresses the benchmark rate of return, where the benchmark is the equally weighted portfolio constructed on the considered market [65]. Then, $\beta^{-}$ and $\beta^{+}$ can be defined as in [66]:

$$\beta^{-}=\frac{Cov(R_{i},R_{B}|R_{B}<\tau )}{Var(R_{B}|R_{B}<\tau )}$$

and

$$\beta^{+}=\frac{Cov(R_{i},R_{B}|R_{B}>\tau )}{Var(R_{B}|R_{B}>\tau )}$$

where τ is the target threshold for the benchmark rate of return. Together, the downside beta and upside beta can be combined by introducing the so-called upside-to-downside beta ratio:

(37) $$U/D\;{\mathrm{Ratio}}_{i}=\frac{\beta_{i}^{+}}{\beta_{i}^{-}}\;.$$

The larger the ratio, the more effectively an asset increases the returns during market upswings, without significantly amplifying the losses during downturns. To compute $\beta^{-}$ and $\beta^{+}$, this study considers the last two years of daily observations, and the threshold τ is set to zero.

### 6.3. Ex Post Performance Metrics

The experimental analysis of this paper has a twofold objective. On the one hand, it investigates whether the inclusion of the risk budgeting constraints improves the control of portfolio risk, by comparing the two proposed asset allocation models. On the other hand, the aim is to analyze the strengths and differences in the two weighting schemes described in Section 4.1 for the TODIM procedure, namely the equal weighting and entropy weighting methods. Several ex post metrics are used to evaluate the financial performance of the compared portfolio strategies, and they are divided into two groups, namely risk measures and performance measures. Let $r_{p,t}^{out}=r_{t}^{⊤}x_{t}$ be the realized portfolio rate of return of a given strategy at the end of month *t*, with t=1,…,T (in our case, T=94). Given initial capital $W_{0}$, the wealth at the end of investing period *t* is $W_{t}=W_{t-1}\;\left(r_{p,t}^{out}+1\right)$, for t=1,⋯,T, where $W_{t-1}$ is its amount in the previous month.

After defining this quantity, the capacity of an investment strategy to avoid high losses through the drawdown risk measure is given by

(38) $$DD_{t}=\min\left\{0,\frac{W_{t}-W_{peak}}{W_{peak}}\right\}$$

where $W_{peak}$ is the maximum amount of wealth reached by the strategy by the end of month *t*. Then, two ex post risk measures linked to drawdowns are considered, namely the maximum,

(39) $$maxDD=\max_{t=1,\cdots ,T}-DD_{t}\;,$$

and the Ulcer index,

(40) $$UI=\sqrt{\frac{\sum_{t=1}^{T}DD_{t}^{2}}{T}}.$$

In particular, the latter evaluates the depth and the duration of drawdowns in wealth over the out-of-sample period [67]. Note that a smaller value for the three metrics indicates better control of the drawdown risk.

Regarding the performance metrics, to evaluate the attractiveness of the proposed investment strategies, the so-called compound annual growth rate (shortly, CAGR) is introduced, and it is defined as follows:

(41) $$CAGR={\left(\frac{W_{T}}{W_{0}}\right)}^{\frac{12}{T}}-1.$$

Moreover, we introduce the out-of-sample monthly rate of return and standard deviation, $\mu^{out}$ and $\sigma^{out}$,

$$\mu^{out}=\frac{1}{T}\sum_{t=1}^{T}r_{p,t}^{out},$$

$$\sigma^{out}=\sqrt{\frac{1}{T-1}\sum_{t=1}^{T}{\left(r_{p,t}^{out}-\mu^{out}\right)}^{2}}\;,$$

and calculate the ex post Sharpe ratio, which is defined as the reward compensation per unit of risk, where the standard deviation is used to quantify the risk:

(42) $$SR^{out}=\frac{\mu^{out}-r_{f}}{\sigma^{out}}$$

with $r_{f}$ being the risk-free rate of return, which we set equal to zero. The second considered performance metric is the Sortino–Satchell ratio [68], which is based on the idea that investors are only concerned about the downside part of the risk, the so-called (negative) semi-standard deviation:

$$\sigma_{-}^{out}=\sqrt{\frac{\sum_{t=1}^{T}{\left({\left(r_{p,t}^{out}-\mu^{out}\right)}_{-}\right)}^{2}}{\sum_{t=1}^{T}1_{\left\{r_{p,t}^{out}-\mu^{out}<0\right\}}}}\;,$$

where ${\left(r_{p,t}^{out}-\mu^{out}\right)}_{-}=\min\left\{r_{p,t}^{out}-\mu^{out},0\right\}$, and $1_{A}$ denotes the indicator function of *A*. Thus, the Sortino–Satchell ratio is as follows:

(43) $$SSR^{out}=\frac{\mu^{out}-r_{f}}{\sigma_{-}^{out}}$$

The third employed risk-adjusted performance measure is the Omega ratio [69], a practical tool to establish whether an investment is more likely to be profitable than loss-making. This quantity is calculated as the ratio between out-of-sample profits and losses:

(44) $$\Omega^{out}=\frac{\sum_{t=1}^{T}\left(r_{p,t}^{out}\;1_{\left\{r_{p,t}^{out}>0\right\}}\right)}{-\sum_{t=1}^{T}\left(r_{p,t}^{out}1_{\left\{r_{p,t}^{out}<0\right\}}\right)}\;.$$

### 6.4. Compared Strategies and Benchmark Portfolios

This section introduces the compared investment strategies according to the following notation, which depends on the cardinality size *K* and the risk parity deviation ν.

- ModSharpe-Equi-TODIM_*K*_: the portfolio model (16) that maximizes the modified Sharpe ratio with cardinality K and using the equal weighting method.
- ModSharpe-Entr-TODIM_*K*_: the portfolio model (16) that maximizes the modified Sharpe ratio with cardinality K and using the entropy weighting method.
- ModSharpe-RB-Equi-TODIM_*K*,*ν*_: the proposed risk budgeting portfolio model (17) with cardinality K, risk parity deviation ν, and adopting the equal weighting method.
- ModSharpe-RB-Entr-TODIM_*K*,*ν*_: the proposed risk budgeting portfolio model (17) with cardinality K, risk parity deviation ν, and adopting the entropy weighting method.

Moreover, the following two benchmark strategies are considered.

- Bench_*EW*_: the equally weighted portfolio constructed using all assets in the investable universe.
- Bench_*MI*,*K*_: an equally weighted strategy that adopts a preliminary stock-picking technique only based on the mutual information criterion for each of the three choices for *K*.

### 6.5. Discussion of the Ex Post Investment Results

Table 2 presents the ex post results of the proposed automated decision support system for the two problem instances, compared with the benchmark strategies introduced in Section 6.4, using the US data set. Observe that, for K=5%, the models employing the entropy weighting method in the TODIM procedure generally display higher risk-adjusted performance ratios. Among these strategies, those based on risk budgeting perform better than their counterparts that do not incorporate risk control. Conversely, better results in terms of risk measures are obtained when employing the equally weighted method in TODIM. It is worth noting that the three risk budgeting models display better control of portfolio volatility and limited drawdowns. This evidence suggests that adapting the criteria weights according to market signals is beneficial for performance, albeit at the cost of higher volatility and greater loss exposure during market downswings. Lastly, note that the mutual information benchmark excels in terms of risk control and also demonstrates competitive risk-adjusted performance. Furthermore, expanding the number of portfolio constituents enhances the ex post results across many of the examined models. For the case K=10%, portfolio models with risk budgeting restrictions that employ equal criteria weighting display significantly better results than in the previous case. In detail, these three models show higher ex post risk-adjusted performance ratios, diminished ex post standard deviations, and lower maximum drawdowns and Ulcer index values. Strategies that use the entropy weighting method yield analogous outcomes to the aforementioned case. For the cardinality K=15%, there is solid evidence of improvement for the entropic-based investments. More precisely, the ModSharpe-Entr-TODIM_15%_ strategy shows very high risk-adjusted performance and good results in terms of risk measures, being the only tested model that is capable of outperforming the equally weighted benchmark. In order to assess whether the differences in performance are statistically significant, a robustness check on these results is conducted. The idea is to test the hypothesis that the out-of-sample Sharpe ratios of the compared strategies are equal. To do this, this work considers the approach introduced in [70], which is based on a circular block bootstrap approach with 5000 bootstrap resamples and automatic optimal block length determination. Due to their size, the tables reporting these comparisons are included in Appendix D, where Table A1 depicts the US case study. The results in terms of the *p*-values for tests in which the null hypothesis is that the Sharpe ratio difference between two compared models is zero are displayed. Specifically, in instances where the null hypothesis is rejected (the observed *p*-value is lower than 0.05), the alternative hypothesis that is considered is consistent with the observed difference between the two Sharpe ratios. A ‘+’ sign is used to denote a positive difference, indicating that the model in the rows outperforms the one in the columns, while a ‘-’ is used to denote a negative difference, indicating that the model in the columns outperforms the one in the rows. Moreover, the false discovery rate approach proposed in [71] is used to determine the proportions of over-, equal, and under-performing methods, in terms of the Sharpe ratio, between all compared ones. To perform these analyses, the RStudio package PeerPerformance [72] (https://CRAN.R-project.org/package=PeerPerformance, accessed on 17 April 2025) has been exploited. This table points out that the ModSharpe-Entr-TODIM_15%_ strategy is statistically superior to the majority of the other analyzed strategies in terms of the Sharpe ratio. The other portfolio models that, according to the adopted method, are more likely to be the over-performing ones are the equally weighted benchmark and MSR-RB-Entr-TODIM_15%,*ν*_. In all other cases, the test for differences in the Sharpe ratios does not show statistically significant results.

**Figure 2:**
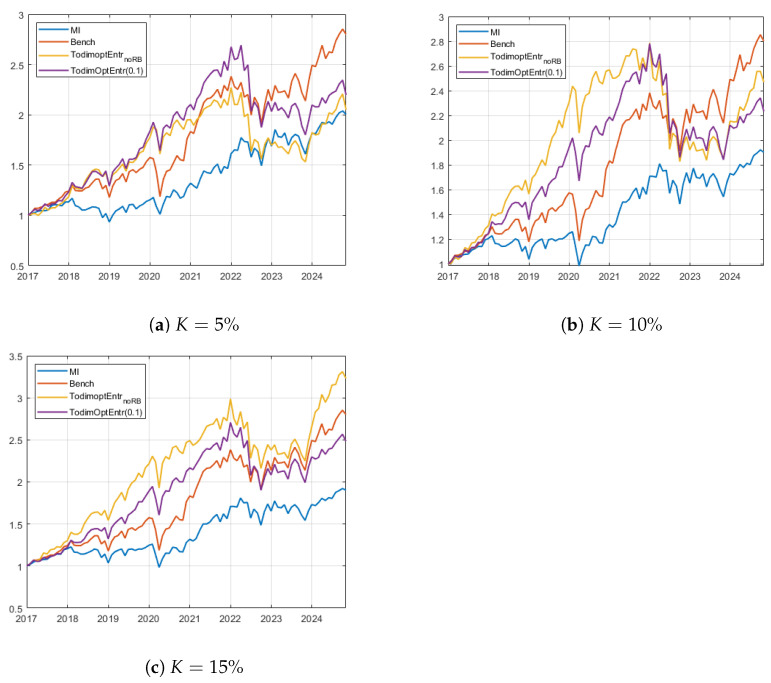
Wealth evolution for the best selected strategies corresponding to the best ex post models (MSR-Entr-TODIM_*K*_ and MSR-RB-Entr-TODIM_*K*,0.1_) and the benchmarks for the US case study. Panel (**a**) shows results for K=5%, while panels (**b**) and (**c**) display K=10% and K=15%, respectively.

Given these considerations, Figure 2 displays the equity lines of the strategies MSR-Entr-TODIM_*K*_ and MSR-RB-Entr-TODIM_*K*,0.1%_ during the investment phase for the US case study, for the three values of *K*. At first, we observe that the equally weighted US market benchmark shows competitive results, almost increasing the initial wealth allocation threefold. In particular, this strategy exhibits elevated performance spikes after the COVID-19 outbreak and during the last two years. Panels (a) and (b) display the results for the K=5% and K=10% cases. Notice that the proposed portfolio models are subject to a marked drawdown phase during the first quarter of 2022 after being the best-performing investment plans during the first five years of allocation. This issue is due to the high volatility of the markets at this time, caused by the aftermath of the COVID-19 pandemic and the international tension brought about by the Russian invasion of Ukraine. Conversely, this drawdown is much less pronounced in the two benchmarks due to the higher diversification of the EW benchmark and a more loss-mitigating stock selection made by the mutual information-based model. Moreover, after this downturn, the two portfolios that maximize the modified Sharpe ratio struggle to regain ground, while the EW benchmark shows positive momentum until the conclusion of the investment period. In the 15% cardinality case, the proposed portfolio strategies more efficiently curtail losses during market downturns. This assertion is further substantiated by Table 2, which shows that the two strategies under consideration display lower maximum drawdowns and Ulcer index values within this cardinality configuration. To conclude, despite its underperformance in terms of produced wealth, the mutual information-based benchmark strategy demonstrates superior loss and volatility control in all analyzed cases.

The results for the EU case study are displayed in Table 3. The European market findings deviate considerably from the American market case. Firstly, strategies based on the entropic weighting method to determine the criteria preferences demonstrate markedly inferior results compared to their counterparts that utilize the equal weighting method. In contrast to the observations made in the US case study, adapting the relative preferences on the three criteria based on market information results in a deterioration in both the risk-adjusted performance ratios and risk control measures.

To assess these considerations in terms of out-of-sample Sharpe ratios, statistical tests on the differences are performed, as seen in Table A1. The test results indicate that MSR-Entr-TODIM_5%_ exhibits the least efficacy, as evidenced by its significantly lower Sharpe ratio in comparison to numerous alternative strategies. In addition, as commented previously, strategies that adopt entropic weights demonstrate marked discrepancies (in negative terms) in comparison to their counterparts that employ the equally weighted method.

The mutual information-based benchmark strategies yield highly competitive outcomes regarding financial performance and show a good capacity to control the ex post standard deviation and drawdown measures. Among all the analyzed allocation plans, the MSR-Equi-TODIM_*K*_ configurations without risk budgeting constraints are the most successful in every respect, producing results aligned with those of the MI-based and the equally weighted benchmarks.

Regarding the statistical tests on the Sharpe ratio differences, the results in Table A1 confirm these insights. Observe that the MI-based benchmarks are the ones with the greatest over-performance, together with the Bench_*EW*_ strategy. Moreover, the MSR-Equi-TODIM_5%_ configurations are characterized by high $p_{+}$ values, meaning that they are likely to be in the group of the best-performing strategies. Finally, it is correct to note that (i) there are no significant differences in the group of entropic portfolio strategies and (ii) despite being the strategy with the highest value of $p_{+}$ detected, Bench_*MI*,5%_ shows statistically significant differences only against a few of the alternatives in the pairwise comparisons. In this data set, incorporating the risk budgeting constraints within the model during the portfolio construction phase does not enhance the financial performance or the portfolio loss control. In the cases of K=5% and K=10%, the optimal parameter value for the parameter ν appears to be 0.05, while, when the cardinality is 15%, the most favorable outcomes are obtained with ν=0.1.

**Figure 3:**
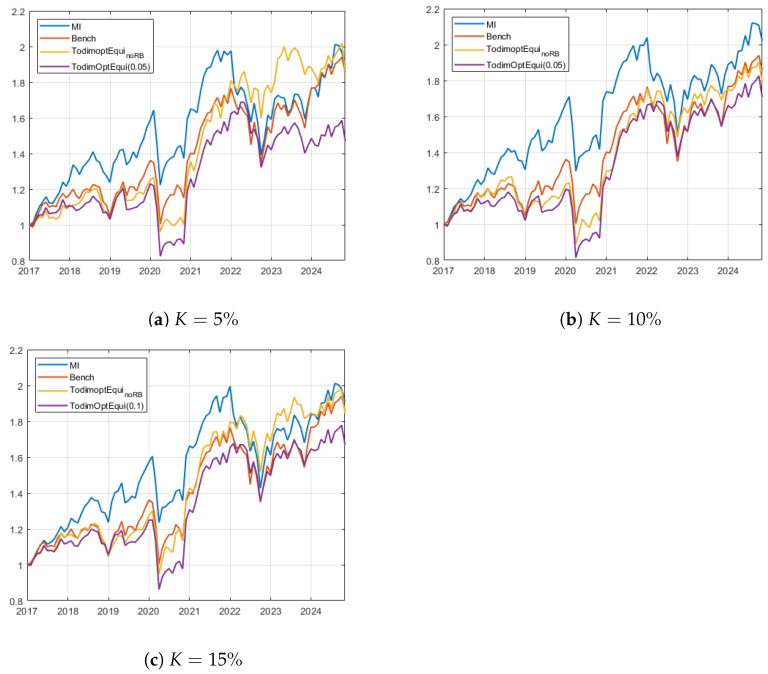
Wealth evolution for the best selected strategies corresponding to the best ex post models and the benchmarks for the EU case study. Panel (**a**) shows the cardinality K=5% and the strategies MSR-Equi-TODIM_5%_ and MSR-RB-Equi-TODIM_5%,0.05_. Panel (**b**) displays the results for the strategies MSR-Equi-TODIM_15%_ and MSR-RB-Equi-TODIM_10%,0.05_. Finally, graph (**c**) shows the evolution of MSR-Equi-TODIM_15%_ and MSR-RB-Equi-TODIM_15%,0.1_.

Figure 3 shows the equity lines of the best-performing strategies and the benchmarks for the EU case study. Panels (a) and (b) illustrate the strategies MSR-Equi-TODIM_*K*_ and MSR-RB-Equi-TODIM_*K*,0.05_ for the cardinalities K=5% and K=10%. Panel (c) shows the evolution of MSR-Equi-TODIM_*K*_ and MSR-RB-Equi-TODIM_*K*,0.1_ for K=15%. In this case study, it is evident that the mutual information-based benchmark emerges as the optimal investment strategy, particularly in the context of K=10%. In the K=5% scenario, the MSR-Equi-TODIM_5%_ portfolio demonstrates the highest equity line at the end of the investment period, despite a turbulent phase during the year 2023. Conversely, the risk budgeting portfolio struggles to recover after periods of market decline, as observed in the post-pandemic era and during the final two years of the investment period. In the K=10% setting, the modified Sharpe-based portfolios prove ineffective in surpassing the MI-based benchmark, yielding outcomes comparable to those of the equally weighted European market benchmark. In the last case (K=15%), the MSR-Equi-TODIM_15%_ strategy demonstrates results aligned with the two benchmarks, while the risk budgeting model shows marginally diminished performance in terms of wealth generation capabilities.

## 7. Conclusions

This study proposes a novel knowledge-based system built upon two interconnected modules, grounded in information theory and financial practices, to assist investors in their financial decision-making. In this context, two instances of a constrained portfolio selection model where the objective function to optimize is a modified version of the Sharpe ratio have been addressed. The first one considers several standard constraints encompassing cardinality, budget, and bound limitations. The second introduces a relaxed risk parity constraint to explicitly control the portfolio volatility in the construction phase. Moreover, a stock-picking procedure that uses a multi-criteria decision-making method named TODIM, which has gained widespread popularity over the years, deals with the cardinality requirement. This technique exploits information from a complementary set of three financial criteria: the mutual information-based peripherality measure, momentum, and the upside-to-downside beta ratio. Finally, a version of the recently proposed distance-based success history differential evolution with double crossover (DISH-XX) algorithm, equipped with an ensemble of constraint-handling techniques, solves the proposed portfolio selection models.

The following summarizes the main experimental findings of this paper. Firstly, the proposed automated decision support system has proven capable of supporting the investment choices of an end user whose preferences are outlined by the two portfolio models analyzed in this paper. Implementing the TODIM module to screen stocks based on three complementary criteria improves the adaptability to the two market scenarios considered. The mutual information-based stock-picking strategy achieves excellent results, especially in a resilient market like the European one, efficiently capturing non-linear relationships between stocks and the market microstructure. This allows potential investors to contain losses during market downswings. Additionally, incorporating criteria such as momentum and the upside-to-downside beta ratio enhances the model’s adaptability to market phases and leverages potential upswings in a thriving market like the American one. Moreover, regarding the optimization module, the solving capabilities of the proposed evolutionary algorithm have been analyzed, demonstrating the convergence of the provided solutions toward the feasible region and the efficient exploration of the search space.

In the subsequent phase of the experimental analysis, this study assessed the financial performance of the proposed investment models, implementing an investment plan with monthly rebalancing from January 2017 to October 2024. Specifically, we compared our strategies against an equally weighted benchmark in both the American and European markets, as well as a mutual information-based stock-picking strategy. The results varied significantly between the two case studies. In the American market, considering a portfolio with 15% of the investable universe resulted in an enhancement in the risk-adjusted performance. In particular, the model without risk budgeting constraints is the only one that can outperform the equally weighted benchmark. In the European case study, the proposed strategy mimics more efficiently the behavior of the equally weighted benchmark and achieves results comparable to those of the mutual information-based benchmark, which is the best-performing portfolio model in the EU case.

A possible limitation of this research is that it adopts a limited number of features for the stock selection phase. Indeed, it would be beneficial to incorporate additional types of information as criteria, such as technical indicators or fundamental analysis metrics extrapolated from firms’ balance sheets. To extend the topic in the field of sustainability (especially relevant in the European context), non-financial disclosure information could also be included as a discriminant. Furthermore, another limitation is represented by the choice of the weighting methods, as we were limited to comparing the performance of only two techniques.

This paper lays the groundwork for several possible future research directions. The first possibility is to consider additional techniques that account not only for agent preferences but also for the predictive capacity of individual criteria over time. Moreover, the flexibility of the proposed knowledge-based financial management system can be tested by considering alternative portfolio models that maximize different objective functions, thereby outlining various investment profiles. To further enhance the practical relevance of the model, an additional constraint to control transaction costs during the investment phase can be introduced. Finally, a third possible extension involves applying different metaheuristics to identify the most suitable algorithm for our cardinality-constrained portfolio optimization models.

## Figures and Tables

**Table 1:** Summary of the considered data sets and experimental design.

Data Set	*n*	Time Window	Estimation Window (Months)	Ex Post Months
S&P 500 (US)	470 stocks	31/12/2014–31/10/2024	24	94
STOXX Europe 600 (EU)	535 stocks	31/12/2014–31/10/2024	24	94

**Table 2:** Ex post results for the two Sharpe ratio-based optimization strategies in the US data set, compared with the benchmark strategies. The first two columns report the value of the fraction $K_{\%}$ of assets comprising the portfolio and the name of the portfolio strategy, respectively. For simplicity of notation, this table omits the dependence from the parameter *K* of the considered strategies. The other columns show the results of the ex post metrics presented in Section 6.3.

US Data Set								
**Configuration**		*CAGR*	*SR*	*SSR^out^*	Ω^*out*^	*σ^out^* (×100)	*maxDD*	*UI*
K = 5%	MSR-Equi-TODIM	0.08	0.15	0.21	1.49	5.00	0.35	0.14
	MSR-RB-Equi-TODIM_0.01_	0.08	0.16	0.23	1.53	4.94	0.30	0.12
	MSR-RB-Equi-TODIM_0.05_	0.08	0.16	0.23	1.53	4.94	0.30	0.12
	MSR-RB-Equi-TODIM_0.1_	0.08	0.16	0.23	1.53	4.95	0.30	0.12
	MSR-Entr-TODIM	0.10	0.18	0.22	1.61	4.99	0.33	0.13
	MSR-RB-Entr-TODIM_0.01_	0.10	0.19	0.25	1.64	5.03	0.34	0.13
	MSR-RB-Entr-TODIM_0.05_	0.10	0.19	0.25	1.63	5.03	0.34	0.13
	MSR-RB-Entr-TODIM_0.1_	0.11	0.19	0.25	1.64	5.02	0.33	0.13
	Bench_*MI*_	0.09	0.19	0.27	1.62	4.50	0.20	0.06
K = 10%	MSR-Equi-TODIM	0.09	0.18	0.23	1.60	4.49	0.29	0.12
	MSR-RB-Equi-TODIM_0.01_	0.10	0.19	0.25	1.61	4.64	0.27	0.10
	MSR-RB-Equi-TODIM_0.05_	0.10	0.19	0.24	1.61	4.64	0.27	0.10
	MSR-RB-Equi-TODIM_0.1_	0.10	0.19	0.25	1.62	4.64	0.27	0.10
	MSR-Entr-TODIM	0.12	0.22	0.27	1.79	4.97	0.34	0.15
	MSR-RB-Entr-TODIM_0.01_	0.11	0.20	0.25	1.68	4.99	0.34	0.14
	MSR-RB-Entr-TODIM_0.05_	0.11	0.20	0.25	1.67	4.99	0.34	0.14
	MSR-RB-Entr-TODIM_0.1_	0.11	0.20	0.25	1.67	4.99	0.34	0.14
	Bench_*MI*_	0.09	0.19	0.27	1.65	4.54	0.19	0.06
K = 15%	MSR-Equi-TODIM	0.12	0.22	0.29	1.76	4.91	0.33	0.12
	MSR-RB-Equi-TODIM_0.01_	0.10	0.19	0.25	1.64	4.71	0.25	0.09
	MSR-RB-Equi-TODIM_0.05_	0.10	0.19	0.25	1.64	4.70	0.25	0.09
	MSR-RB-Equi-TODIM_0.1_	0.10	0.19	0.25	1.64	4.70	0.25	0.09
	MSR-Entr-TODIM	0.16	0.28	0.34	2.05	4.92	0.28	0.10
	MSR-RB-Entr-TODIM_0.01_	0.12	0.23	0.28	1.80	4.82	0.30	0.11
	MSR-RB-Entr-TODIM_0.05_	0.12	0.23	0.28	1.81	4.81	0.30	0.11
	MSR-RB-Entr-TODIM_0.1_	0.12	0.23	0.28	1.81	4.81	0.30	0.11
	Bench_*MI*_	0.09	0.17	0.25	1.58	4.65	0.22	0.06
Bench_*EW*_		0.14	0.24	0.33	1.89	5.06	0.25	0.06

**Table 3:** Ex post results for the two Sharpe ratio-based optimization strategies in the EU data set, compared with the benchmark strategies. The first two columns report the value of the fraction $K_{\%}$ of assets comprising the portfolio and the portfolio strategy, respectively. For simplicity of notation, this table omits the dependence from the parameter *K* of the considered strategies. The other columns show the results of the ex post metrics presented in Section 6.3.

EU Data Set								
**Configuration**		*CAGR*	*SR*	*SSR^out^*	Ω^*out*^	*σ^out^* (×100)	*maxDD*	*UI*
K = 5%	MSR-Equi-TODIM	0.08	0.16	0.23	1.58	4.93	0.24	0.07
	MSR-RB-Equi-TODIM_0.01_	0.05	0.10	0.13	1.37	5.69	0.33	0.11
	MSR-RB-Equi-TODIM_0.05_	0.05	0.10	0.13	1.38	5.68	0.33	0.11
	MSR-RB-Equi-TODIM_0.1_	0.05	0.10	0.13	1.37	5.66	0.33	0.11
	MSR-Entr-TODIM	0.02	0.06	0.08	1.20	5.97	0.43	0.15
	MSR-RB-Entr-TODIM_0.01_	0.02	0.05	0.07	1.19	6.43	0.43	0.15
	MSR-RB-Entr-TODIM_0.05_	0.02	0.05	0.07	1.19	6.43	0.43	0.15
	MSR-RB-Entr-TODIM_0.1_	0.02	0.05	0.07	1.19	6.40	0.43	0.15
	Bench_*MI*_	0.09	0.17	0.23	1.54	4.79	0.30	0.10
K = 10%	MSR-Equi-TODIM	0.08	0.15	0.21	1.55	4.79	0.30	0.08
	MSR-RB-Equi-TODIM_0.01_	0.07	0.13	0.16	1.50	5.45	0.32	0.09
	MSR-RB-Equi-TODIM_0.05_	0.07	0.13	0.16	1.51	5.44	0.32	0.09
	MSR-RB-Equi-TODIM_0.1_	0.07	0.13	0.16	1.51	5.43	0.32	0.09
	MSR-Entr-TODIM	0.06	0.11	0.13	1.37	5.35	0.39	0.13
	MSR-RB-Entr-TODIM_0.01_	0.04	0.09	0.11	1.32	6.09	0.40	0.12
	MSR-RB-Entr-TODIM_0.05_	0.04	0.09	0.11	1.32	6.08	0.39	0.12
	MSR-RB-Entr-TODIM_0.1_	0.04	0.09	0.11	1.32	6.08	0.40	0.12
	Bench_*MI*_	0.09	0.19	0.25	1.65	4.63	0.26	0.09
K = 15%	MSR-Equi-TODIM	0.08	0.16	0.22	1.58	4.84	0.27	0.07
	MSR-RB-Equi-TODIM_0.01_	0.07	0.13	0.16	1.49	5.28	0.31	0.08
	MSR-RB-Equi-TODIM_0.05_	0.07	0.13	0.16	1.49	5.28	0.31	0.08
	MSR-RB-Equi-TODIM_0.1_	0.07	0.13	0.16	1.49	5.26	0.31	0.08
	MSR-Entr-TODIM	0.05	0.10	0.12	1.33	5.38	0.37	0.11
	MSR-RB-Entr-TODIM_0.01_	0.05	0.10	0.11	1.34	5.81	0.36	0.10
	MSR-RB-Entr-TODIM_0.05_	0.05	0.10	0.11	1.34	5.81	0.36	0.10
	MSR-RB-Entr-TODIM_0.1_	0.05	0.10	0.11	1.35	5.81	0.36	0.10
	Bench_*MI*_	0.09	0.18	0.23	1.60	4.52	0.28	0.09
Bench_*EW*_		0.08	0.16	0.22	1.54	4.80	0.26	0.08

**Figure A1:**
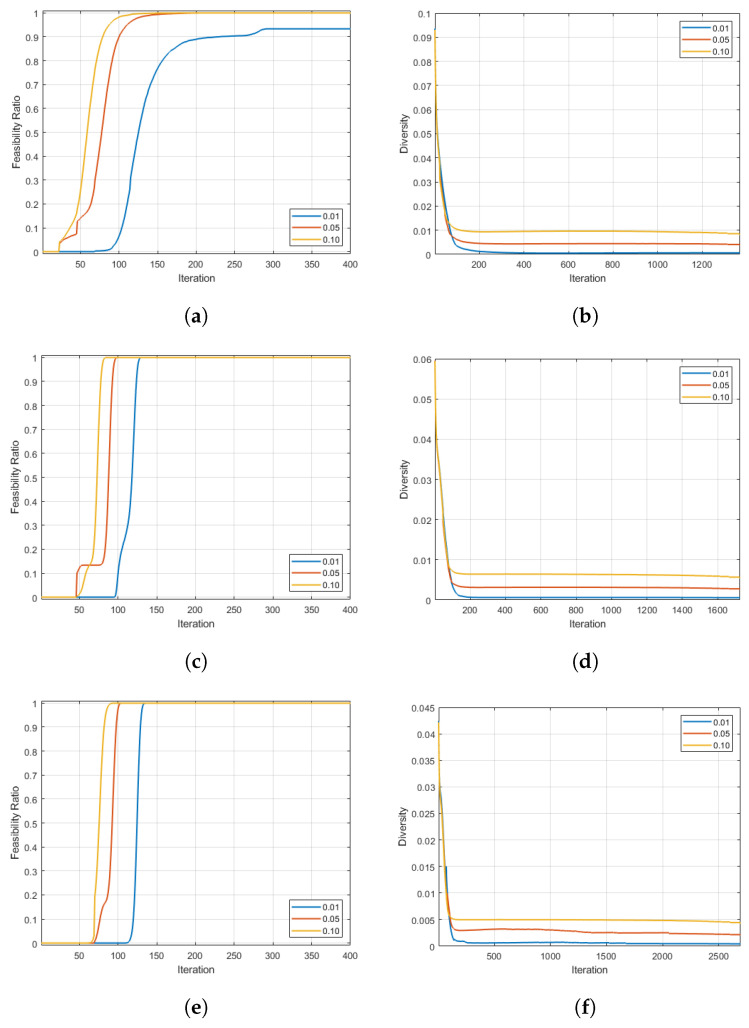
Average results for 30 random initial portfolio configurations of the feasible ratio and the diversity measure for the US case study by varying the deviation parameter ν in {0.01,0.05,0.10}. Plots (**a**,**b**) show the results for K=23 (5%); plots (**c**,**d**) show the results for K=47 (10%). Finally, results for K=70 (15%) are displayed in charts (**e**,**f**).

**Figure A2:**
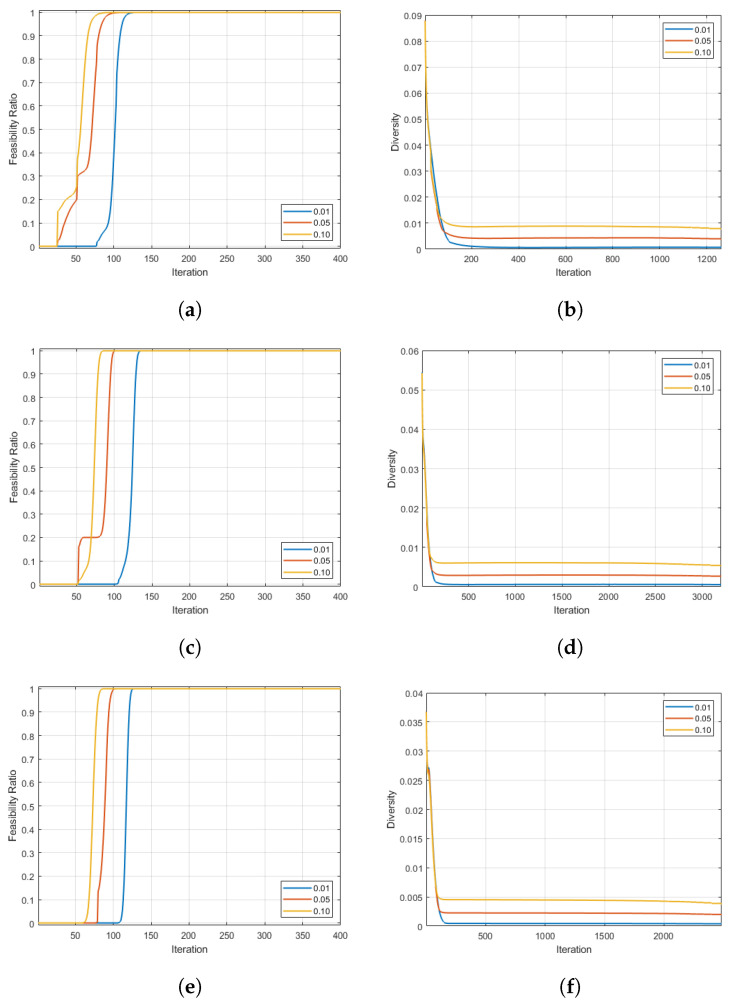
Average results for 30 random initial portfolio configurations of the feasible ratio and the diversity measure for the EU case study by varying the deviation parameter ν in {0.01,0.05,0.10}. Plots (**a**,**b**) show the results for K=26 (5%); plots (**c**,**d**) show the results for K=53 (10%). Finally, results for K=80 (15%) are displayed in charts (**e**,**f**).

## Data Availability

The data sets presented in this article are not readily available because there are technical limitations imposed by the data provider.

## Appendix A. Risk Parity and Risk Budgeting

### Appendix A.1. Details of Risk Parity

The modern risk parity asset allocation framework has recently gained traction in both academia and industry. In this setting, wealth is assigned in such a way that the individual assets’ contributions to portfolio risk are equalized. As shown in [17], this goal can be achieved through the Euler decomposition of the portfolio risk measure, under the condition that the risk measure must be a homogeneous function of degree one. It is readily shown that the portfolio standard deviation meets this requirement. Moreover, this quantity can be decomposed as follows:

(A1) $$\sigma_{p}\left(w\right)=\sum_{i=1}^{n}w_{i}\frac{\partial \sigma_{p}\left(w\right)}{\partial w_{i}}=\sum_{i=1}^{n}\frac{w_{i}{\left(\Sigma w\right)}_{i}}{\sqrt{w^{⊤}\Sigma w}}\;.$$

Given the marginal risk contribution of asset *i*$\frac{\partial \sigma_{p}\left(w\right)}{\partial w_{i}}=\frac{{\left(\Sigma w\right)}_{i}}{\sqrt{w^{⊤}\Sigma w}}$, it follows that $\frac{w_{i}{\left(\Sigma w\right)}_{i}}{\sqrt{w^{⊤}\Sigma w}}=RC_{i}\left(w\right)$ denotes its risk contribution. The corresponding relative risk contribution is defined as

(A2) $$RRC_{i}\left(w\right)=\frac{RC_{i}\left(w\right)}{\sqrt{w^{⊤}\Sigma w}}=\frac{w_{i}{\left(\Sigma w\right)}_{i}}{w^{⊤}\Sigma w}\;.$$

The risk parity framework seeks to compute the portfolio that equalizes all risk contributions, satisfying the condition

(A3) $$w_{i}{\left(\Sigma w\right)}_{i}=w_{j}{\left(\Sigma w\right)}_{j}\;\forall i,j\;.$$

Moreover, in the risk parity portfolio, combining the decomposition introduced in Equation (A1) and the requirement expressed in Equation (A3), it follows that

(A4) $$RC_{i}\left(w\right)=\frac{\sqrt{w^{⊤}\Sigma w}}{n}=\frac{\sigma_{p}\left(w\right)}{n}\;\forall i\;.$$

### Appendix A.2. Non-Convexity of the Proposed Risk Budgeting Formulation

Following [30], $RC_{i}\left(w\right)$ can be recast using the standard matrix notation as

(A5) $$RC_{i}\left(w\right)=\frac{w^{⊤}E_{i}w}{\sqrt{w^{⊤}\Sigma w}}\;\forall i$$

where $E_{i}\in R^{n\times n}$ captures the individual risk contribution of asset *i*. The symmetric matrices $E_{i}$ are composed of the superposition of row *i* and column *i* from the original covariance matrix Σ multiplied by one half, with all other elements in the matrix equal to zero, according to the following formula:

(A6) $$E_{i}=\frac{1}{2}\left(e_{i}e_{i}^{⊤}\Sigma +\Sigma e_{i}e_{i}^{⊤}\right)$$

where $e_{i}\in R^{n}$ denotes the *i*th column of the identity matrix. Notice that the set of inequalities introduced in Equation (9) can be rewritten as

(A7) $$\left\{\begin{array}{c} \left(1-\underline{\nu }\right)\frac{\sqrt{w^{⊤}\Sigma w}}{n}-\frac{w_{i}{\left(\Sigma w\right)}_{i}}{\sqrt{w^{⊤}\Sigma w}}\leq 0,\;i=1,...,n\\ \frac{w_{i}{\left(\Sigma w\right)}_{i}}{\sqrt{w^{⊤}\Sigma w}}-\left(1+\bar{\nu }\right)\frac{\sqrt{w^{⊤}\Sigma w}}{n}\leq 0,\;i=1,...,n. \end{array}\right.$$

and, with some algebra, the following set of conditions follows:

(A8) $$\left\{\begin{array}{c} w^{⊤}\left(\frac{\left(1-\underline{\nu }\right)}{n}\Sigma -E_{i}\right)w\leq 0,\;i=1,...,n\\ w^{⊤}\left(E_{i}-\frac{\left(1+\bar{\nu }\right)}{n}\Sigma \right)w\leq 0,\;i=1,...,n. \end{array}\right.$$

Since $E_{i}$ is symmetric, the difference $E_{i}-\frac{\left(1+\bar{\nu }\right)}{n}\Sigma$ is still a symmetric matrix. Then, the generic difference is the following, with $\frac{\left(1+\bar{\nu }\right)}{n}=\bar{\gamma }\left(<1\right)$:

$$\left(\begin{array}{cccccc} 0 & 0 & \cdots & \frac{\sigma_{1i}}{2} & \cdots & 0\\ 0 & 0 & \cdots & \frac{\sigma_{2i}}{2} & \cdots & 0\\ \vdots & \vdots & \ddots & \vdots & & \vdots \\ \frac{\sigma_{i1}}{2} & \frac{\sigma_{i2}}{2} & \cdots & \sigma_{i}^{2} & \cdots & \frac{\sigma_{in}}{2}\\ \vdots & \vdots & & \vdots & \ddots & \vdots \\ 0 & 0 & \cdots & \frac{\sigma_{ni}}{2} & \cdots & 0 \end{array}\right)-\bar{\gamma }\left(\begin{array}{cccccc} \sigma_{1}^{2} & \sigma_{12} & \cdots & \sigma_{1i} & \cdots & \sigma_{1n}\\ \sigma_{21} & \sigma_{2}^{2} & \cdots & \sigma_{2i} & \cdots & \sigma_{2n}\\ \vdots & \vdots & \ddots & \vdots & & \vdots \\ \sigma_{i1} & \sigma_{i2} & \cdots & \sigma_{i}^{2} & \cdots & \sigma_{in}\\ \vdots & \vdots & & \vdots & \ddots & \vdots \\ \sigma_{n1} & \sigma_{n2} & \cdots & \sigma_{ni} & \cdots & \sigma_{n}^{2} \end{array}\right),$$

This results in the matrix

$${\bar{\Gamma }}_{i}=\left(\begin{array}{cccccc} -\bar{\gamma }\sigma_{1}^{2} & -\bar{\gamma }\sigma_{12} & \cdots & \frac{\sigma_{1i}}{2}-\bar{\gamma }\sigma_{1i} & \cdots & -\bar{\gamma }\sigma_{1n}\\ -\bar{\gamma }\sigma_{21} & -\bar{\gamma }\sigma_{2}^{2} & \cdots & \frac{\sigma_{2i}}{2}-\bar{\gamma }\sigma_{2i} & \cdots & -\bar{\gamma }\sigma_{2n}\\ \vdots & \vdots & \ddots & \vdots & \; & \vdots \\ \frac{\sigma_{i1}}{2}-\bar{\gamma }\sigma_{i1} & \frac{\sigma_{i2}}{2}-\bar{\gamma }\sigma_{i2} & \cdots & \sigma_{i}^{2}-\bar{\gamma }\sigma_{i}^{2} & \cdots & \frac{\sigma_{in}}{2}-\bar{\gamma }\sigma_{in}\\ \vdots & \vdots & \; & \vdots & \ddots & \vdots \\ -\bar{\gamma }\sigma_{n1} & -\bar{\gamma }\sigma_{n2} & \cdots & \frac{\sigma_{ni}}{2}-\bar{\gamma }\sigma_{ni} & \cdots & -\bar{\gamma }\sigma_{n}^{2} \end{array}\right).$$

Inspecting the matrices ${\bar{\Gamma }}_{i}$ reveals that they are indefinite, each having both positive and negative elements on the diagonal [73]. Thus, any optimization problem that involves the use of ${\bar{\Gamma }}_{i}$ is non-convex. Similar conclusions can be inferred for the matrix obtained from the difference $\frac{\left(1-\underline{\nu }\right)}{n}\Sigma -E_{i}$.

## Appendix B. Pseudocode of DISH-XX-εg

The pseudocode of the proposed DISH-XX-*ε*g is given in Algorithm A1 for the solution of Problem (17).

**Algorithm A1** DISH-XX-εg
**Input: ***K*, μ, Σ, $lb_{i}$ and $ub_{i}$ for i=1,…,K, $g_{j}$ for j=1,…,2K Set H=5, $\alpha_{max}=0.25$, $\alpha_{min}=0.125$, A=∅, $A_{best}=\emptyset$ Initialize the historical memory arrays $M_{F}$ and $M_{Cr}$ using Equations (18) and (19) Calculate $NP_{init}=50\ln\left(K\right)\sqrt{K}$ and $FES_{max}$ using Equation (34) Set $NP=NP_{init}$ FES:=0, t:=0, k:=1 Generate and evaluate the initial population P Update FES Initialize the ε level using Equation (31) Set $x^{best}$ as the best solution in P based on the ε-comparison (29) **while** $FES<FES_{max}$ **do** t:=t+1 Calculate ε using Equation (31) Apply mutation, double crossover, and the repair operator using Equations (20), (21), (26) and (27) Evaluate the new trial population U and update FES **for** p=1 to NP **do** **if** $\phi \left(u_{i}\right)\neq 0\wedge \mathrm{mod}\left(t,K\right)=0\wedge \mathrm{rand}<0.2$ **then** Apply the gradient-based mutation to $u^{i}$ using Equations (32) and (33) **end if** Apply selection phase using Equation (29) Store the best individuals in archive $A_{best}$ and the worst individuals in archive A Update $S_{F}$, $S_{Cr}$ **if** $S_{F}\neq \emptyset \wedge S_{Cr}\neq \emptyset$ **then** Update $M_{F,k}$, $M_{Cr,k}$ k:=k+1 **if** k>H **then** k:=1 **end if** **end if** **end for** Update population size using Equation (25) **if** |P|>NP **then** Sort the individuals in P according to (29) and delete the |P|−NP worst ones **end if** **if** |A|>NP **then** Randomly delete |A|−NP individuals from A **end if** Update $x^{best}$ **end while**

## Appendix C. Assessment of the Algorithm’s Efficiency

The aim of this section is evaluating whether the proposed algorithm is capable of finding feasible solutions to the risk budgeting portfolio optimization problem. To do this, a date from the 94 available in the ex post window is randomly selected and the nine portfolio configurations presented in Section 6.4 are considered. Then, 30 random different initial portfolios to be optimized are sampled, which already respect the cardinality constraint, in order to demonstrate the algorithm’s search capabilities. In this process, two features are considered: feasibility and diversity. The former is quantified by the so-called feasibility ratio, which is the number of feasible solutions relative to the population size at a given iteration; the latter is evaluated by the population diversity [74], which is defined as

$$div\left(t\right)=\frac{1}{N_{pop}\left(t\right)}\sum_{i=1}^{N_{pop}\left(t\right)}\sqrt{\sum_{i=1}^{d}{\left(y_{ji}\left(t\right)-{\bar{y}}_{i}\left(t\right)\right)}^{2}}$$

where *t* is the generation index, $y_{ji}$ is the *i*th component of the *j*-th candidate solution, and ${\bar{y}}_{i}\left(t\right)$ is the mean position of the population in coordinate *i* at generation *t*. Note that div can be interpreted as the average Euclidean distance between a candidate solution and the barycenter of the population of candidate solutions at time *t*.

Figure A1 illustrates the evolution of the average values for the feasibility ratio and population diversity over the generations in the US case study. From panels (a), (c), and (d), observe that the algorithm reaches a 100% feasibility ratio of the population after approximately 400 iterations in all but one case. (The figures plot only the initial 400 iterations to improve the readability of the graphs). The diversity measure plots (b), (d), and (f) point out how the population, at the final stages, narrows down to a very small portion of the search space, assessing the algorithm’s capability to find near-optimal solutions for the optimization problem.

Figure A2 illustrates the same plots for the EU case study, showing similar findings. The results for other random dates from the out-of-sample window are very close to the ones presented in Figure A1 and Figure A2 and thus are omitted.

## Appendix D. Statistical Significance of Differences Among the Sharpe Ratios of Portfolios

This section analyzes the statistical significance of the differences among the Sharpe ratios of the compared portfolio strategies, according to the robustness Sharpe test introduced in [70], for each pair of the competing portfolios. Table A1 reports the results for the US case study, while Table A2 shows the respective counterparts for the European stock market.

**Table A1:** Assessment of the statistical significance of differences among the Sharpe ratios of the competing portfolios according to the Sharpe ratio robustness test for the US data set [70]. The differences are calculated by comparing the model in the rows against the one in the columns. The *p*-values are reported, and values equal to or lower than 5% are highlighted in bold. The alternative hypothesis is consistent with the sign of the observed difference (‘+’ denotes a positive difference, ‘−’ a negative one). In the bottom part of the table, the proportions of over- ($p_{+}$), equal ($p_{0}$), and under-performing ($p_{-}$) strategies, in terms of the Sharpe ratio, are computed following the approach of [71].

	Bench_*EW*_	Bench_*MI*,5%_	Bench_*MI*,10%_	Bench_*MI*,15%_	Equi-NoRB_5%_	Equi-NoRB_10%_	Equi-NoRB_15%_	Entr-NoRB_5%_	Entr-NoRB_10%_	Entr-NoRB_15%_
Bench_*MI*,5%_	0.4094									
Bench_*MI*,10%_	0.2394	0.9758								
Bench_*MI*,15%_	0.0665	0.6023	0.3479							
Equi-NoRB_5%_	0.1915	0.6093	0.5634	0.7518						
Equi-NoRB_10%_	0.3769	0.9258	0.9178	0.8843	0.3474					
Equi-NoRB_15%_	0.6828	0.6943	0.6488	0.4919	0.1680	0.3219				
Entr-NoRB_5%_	0.3074	0.8718	0.8818	0.9383	0.5559	0.9123	0.4594			
Entr-NoRB_10%_	0.7013	0.7608	0.7198	0.5759	0.1880	0.4649	0.9903	0.2569		
Entr-NoRB_15%_	0.5504	0.2644	0.1585	0.0995	$0.0175^{+}$	$0.0385^{+}$	0.1680	$0.0045^{+}$	$0.0500^{+}$	
Equi-RB_5%,0.01_	0.1910	0.6478	0.5794	0.8238	0.7603	0.5949	0.2639	0.7608	0.3404	$0.0315^{-}$
Equi-RB_5%,0.05_	0.1910	0.6483	0.5749	0.8208	0.7583	0.5859	0.2564	0.7588	0.3354	$0.0315^{-}$
Equi-RB_5%,0.10_	0.1935	0.6448	0.5784	0.8223	0.7433	0.5934	0.2539	0.7553	0.3334	$0.0320^{-}$
Equi-RB_10%,0.01_	0.3634	0.9818	0.9683	0.7818	0.2964	0.8713	0.4799	0.8388	0.5664	0.0575
Equi-RB_10%,0.05_	0.3579	0.9743	0.9583	0.7973	0.3074	0.8853	0.4674	0.8548	0.5544	0.0580
Equi-RB_10%,0.10_	0.3719	0.9858	0.9868	0.7763	0.2819	0.8513	0.4899	0.8323	0.5754	0.0600
Equi-RB_15%,0.01_	0.2514	0.9793	0.9633	0.6853	0.3414	0.8348	0.5019	0.7893	0.6128	$0.0350^{-}$
Equi-RB_15%,0.05_	0.2514	0.9848	0.9663	0.6838	0.3394	0.8338	0.4974	0.7888	0.6103	$0.0350^{-}$
Equi-RB_15%,0.10_	0.2584	0.9778	0.9573	0.6828	0.3349	0.8283	0.5029	0.7838	0.6168	$0.0345^{-}$
Entr-RB_5%,0.01_	0.4014	0.9773	0.9788	0.7748	0.3944	0.8633	0.6063	0.6648	0.5584	$0.0395^{-}$
Entr-RB_5%,0.05_	0.3964	0.9848	0.9853	0.7828	0.4034	0.8763	0.5959	0.6838	0.5469	$0.0380^{-}$
Entr-RB_5%,0.10_	0.4114	0.9678	0.9628	0.7648	0.3819	0.8503	0.6168	0.6368	0.5694	$0.0440^{-}$
Entr-RB_10%,0.01_	0.4254	0.9418	0.9203	0.7233	0.3229	0.7533	0.6668	0.5779	0.5544	$0.0140^{-}$
Entr-RB_10%,0.05_	0.4214	0.9468	0.9243	0.7293	0.3264	0.7593	0.6593	0.5839	0.5464	$0.0140^{-}$
Entr-RB_10%,0.10_	0.4289	0.9448	0.9198	0.7253	0.3239	0.7508	0.6643	0.5779	0.5474	$0.0130^{-}$
Entr-RB_15%,0.01_	0.6988	0.6088	0.5154	0.3649	0.1310	0.3604	0.8828	0.2089	0.8828	0.1100
Entr-RB_15%,0.05_	0.7093	0.6033	0.5129	0.3604	0.1270	0.3544	0.8758	0.2024	0.8743	0.1125
Entr-RB_15%,0.10_	0.7198	0.5994	0.5034	0.3549	0.1270	0.3479	0.8658	0.1980	0.8568	0.1175
$p_{0}$	0.4444	1.0000	1.0000	1.0000	0.4321	1.0000	0.6240	1.0000	0.6522	0.0000
$p_{+}$	0.5556	0.0000	0.0000	0.0000	0.0000	0.0000	0.3760	0.0000	0.3478	1.0000
$p_{-}$	0.0000	0.0000	0.0000	0.0000	0.5679	0.0000	0.0000	0.0000	0.0000	0.0000
	Equi-RB_5%,0.01_	Equi-RB_5%,0.05_	Equi-RB_5%,0.10_	Equi-RB_10%,0.01_	Equi-RB_10%,0.05_	Equi-RB_10%,0.10_	Equi-RB_15%,0.01_	Equi-RB_15%,0.05_	Equi-RB_15%,0.10_	
Bench_*MI*,5%_										
Bench_*MI*,10%_										
Bench_*MI*,15%_										
Equi-NoRB_5%_										
Equi-NoRB_10%_										
Equi-NoRB_15%_										
Entr-NoRB_5%_										
Entr-NoRB_10%_										
Entr-NoRB_15%_										
Equi-RB_5%,0.01_										
Equi-RB_5%,0.05_	0.8633									
Equi-RB_5%,0.10_	0.9353	0.9768								
Equi-RB_10%,0.01_	0.1985	0.1910	0.1895							
Equi-RB_10%,0.05_	0.2159	0.2119	0.2104	0.3524						
Equi-RB_10%,0.10_	0.1850	0.1815	0.1775	0.5739	0.2729					
Equi-RB_15%,0.01_	0.3134	0.3164	0.3149	0.8973	0.8738	0.9283				
Equi-RB_15%,0.05_	0.3164	0.3184	0.3199	0.9053	0.8818	0.9353	0.8453			
Equi-RB_15%,0.10_	0.3044	0.3014	0.3069	0.8838	0.8573	0.9108	0.7473	0.7063		
Entr-RB_5%,0.01_	0.4429	0.4414	0.4374	0.9418	0.9278	0.9528	0.9968	0.9988	0.9853	
Entr-RB_5%,0.05_	0.4494	0.4489	0.4484	0.9513	0.9338	0.9673	0.9853	0.9853	0.9753	
Entr-RB_5%,0.10_	0.4309	0.4279	0.4274	0.9203	0.9008	0.9363	0.9863	0.9843	0.9938	
Entr-RB_10%,0.01_	0.3829	0.3744	0.3784	0.8063	0.7888	0.8238	0.8853	0.8773	0.8938	
Entr-RB_10%,0.05_	0.3889	0.3869	0.3869	0.8158	0.7978	0.8323	0.8978	0.8903	0.9088	
Entr-RB_10%,0.10_	0.3804	0.3754	0.3774	0.8013	0.7853	0.8173	0.8848	0.8813	0.8908	
Entr-RB_15%,0.01_	0.1090	0.1100	0.1075	0.2459	0.2379	0.2584	0.2674	0.2664	0.2674	
Entr-RB_15%,0.05_	0.1085	0.1065	0.1055	0.2389	0.2324	0.2539	0.2604	0.2544	0.2594	
Entr-RB_15%,0.10_	0.1065	0.1055	0.1060	0.2349	0.2299	0.2479	0.2509	0.2494	0.2489	
$p_{0}$	0.5931	0.5931	0.5931	1.0000	1.0000	1.0000	1.0000	1.0000	1.0000	
$p_{+}$	0.0000	0.0000	0.0000	0.0000	0.0000	0.0000	0.0000	0.0000	0.0000	
$p_{-}$	0.4069	0.4069	0.4069	0.0000	0.0000	0.0000	0.0000	0.0000	0.0000	
	Entr-RB_5%,0.01_	Entr-RB_5%,0.05_	Entr-RB_5%,0.10_	Entr-RB_10%,0.01_	Entr-RB_10%,0.05_	Entr-RB_10%,0.10_	Entr-RB_15%,0.01_	Entr-RB_15%,0.05_	Entr-RB_15%,0.10_	
Bench_*MI*,5%_										
Bench_*MI*,10%_										
Bench_*MI*,15%_										
Equi-NoRB_5%_										
Equi-NoRB_10%_										
Equi-NoRB_15%_										
Entr-NoRB_5%_										
Entr-NoRB_10%_										
Entr-NoRB_15%_										
Equi-RB_5%,0.01_										
Equi-RB_5%,0.05_										
Equi-RB_5%,0.10_										
Equi-RB_10%,0.01_										
Equi-RB_10%,0.05_										
Equi-RB_10%,0.10_										
Equi-RB_15%,0.01_										
Equi-RB_15%,0.05_										
Equi-RB_15%,0.10_										
Entr-RB_5%,0.01_										
Entr-RB_5%,0.05_	0.5784									
Entr-RB_5%,0.10_	0.6583	0.5294								
Entr-RB_10%,0.01_	0.8238	0.7973	0.8568							
Entr-RB_10%,0.05_	0.8373	0.8103	0.8643	0.6548						
Entr-RB_10%,0.10_	0.8153	0.7948	0.8523	0.9558	0.7328					
Entr-RB_15%,0.01_	0.1935	0.1865	0.2124	$0.0280^{+}$	$0.0275^{+}$	$0.0260^{+}$				
Entr-RB_15%,0.05_	0.1825	0.1785	0.1995	0.0240	$0.0235^{+}$	$0.0220^{+}$	0.4324			
Entr-RB_15%,0.10_	0.1775	0.1750	0.1960	$0.0230^{+}$	$0.0225^{+}$	$0.0205^{+}$	0.2064	0.5064		
$p_{0}$	1.0000	1.0000	1.0000	1.0000	1.0000	1.0000	0.2963	0.2778	0.2778	
$p_{+}$	0.0000	0.0000	0.0000	0.0000	0.0000	0.0000	0.6741	0.7222	0.7222	
$p_{-}$	0.0000	0.0000	0.0000	0.0000	0.0000	0.0000	0.0296	0.0000	0.0000	

**Table A2:** Assessment of the statistical significance of differences among the Sharpe ratios of the competing portfolios according to the Sharpe ratio robustness test for the EU data set [70]. The differences are calculated by comparing the model in the rows against the one in the columns. The *p*-values are reported, and values equal to or lower than 5% are highlighted in bold. The alternative hypothesis is consistent with the sign of the observed difference (‘+’ denotes a positive difference, ‘−’ a negative one). In the bottom part of the table, the proportions of over- ($p_{+}$), equal ($p_{0}$), and under-performing ($p_{-}$) strategies, in terms of the Sharpe ratio, are computed following the approach of [71].

	Bench_*EW*_	Bench_*MI*,5%_	Bench_*MI*,10%_	Bench_*MI*,15%_	Equi-NoRB_5%_	Equi-NoRB_10%_	Equi-NoRB_15%_	Entr-NoRB_5%_	Entr-NoRB_10%_	Entr-NoRB_15%_
Bench_*MI*,5%_	0.9568									
Bench_*MI*,10%_	0.6213	0.4944								
Bench_*MI*,15%_	0.7688	0.7833	0.5334							
Equi-NoRB_5%_	0.9823	0.9403	0.6898	0.7998						
Equi-NoRB_10%_	0.8613	0.8463	0.5959	0.7188	0.8678					
Equi-NoRB_15%_	0.9423	0.9293	0.6873	0.7923	0.9778	0.8448				
Entr-NoRB_5%_	0.0645	0.1220	$0.0400^{-}$	$0.0470^{-}$	$0.0170^{-}$	$0.0180^{-}$	$0.0225^{-}$			
Entr-NoRB_10%_	0.2994	0.4014	0.2039	0.2629	0.2399	0.2574	0.2089	0.0825		
Entr-NoRB_15%_	0.1115	0.3034	0.1540	0.1830	0.1710	0.0935	$0.0420^{-}$	0.3109	0.6698	
Equi-RB_5%,0.01_	0.1990	0.3114	0.1535	0.1700	0.1100	0.2119	0.1415	0.1630	0.7458	0.9348
Equi-RB_5%,0.05_	0.2059	0.3144	0.1580	0.1730	0.1145	0.2154	0.1475	0.1485	0.7638	0.9143
Equi-RB_5%,0.10_	0.2034	0.3114	0.1525	0.1705	0.1075	0.2089	0.1425	0.1575	0.7503	0.9293
Equi-RB_10%,0.01_	0.3914	0.5784	0.3249	0.3844	0.4699	0.5069	0.4094	$0.0150^{+}$	0.5529	0.2809
Equi-RB_10%,0.05_	0.4099	0.5919	0.3329	0.3949	0.4854	0.5364	0.4304	$0.0145^{+}$	0.5249	0.2589
Equi-RB_10%,0.10_	0.3969	0.5804	0.3284	0.3889	0.4744	0.5114	0.4104	$0.0155^{+}$	0.5404	0.2659
Equi-RB_15%,0.01_	0.3459	0.5484	0.3154	0.3799	0.4654	0.4934	0.2954	$0.0265^{+}$	0.6028	0.2634
Equi-RB_15%,0.05_	0.3469	0.5504	0.3169	0.3819	0.4629	0.4914	0.2924	$0.0270^{+}$	0.6033	0.2619
Equi-RB_15%,0.10_	0.3594	0.5589	0.3259	0.3889	0.4769	0.5119	0.3084	$0.0255^{+}$	0.5809	0.2449
Entr-RB_5%,0.01_	$0.0485^{-}$	0.1335	$0.0480^{-}$	0.0565	$0.0325^{-}$	0.0510	$0.0365^{-}$	0.8658	0.1265	0.3034
Entr-RB_5%,0.05_	$0.0480^{-}$	0.1310	$0.0465^{-}$	0.0555	$0.0315^{-}$	$0.0470^{-}$	$0.0355^{-}$	0.8448	0.1180	0.2939
Entr-RB_5%,0.10_	$0.0460^{-}$	0.1320	$0.0455^{-}$	0.0550	$0.0315^{-}$	$0.0470^{-}$	$0.0325^{-}$	0.8478	0.1185	0.2924
Entr-RB_10%,0.01_	0.1005	0.2669	0.1210	0.1435	0.1470	0.1315	0.0930	0.2644	0.4269	0.7913
Entr-RB_10%,0.05_	0.0995	0.2644	0.1185	0.1410	0.1410	0.1290	0.0905	0.2699	0.4154	0.7813
Entr-RB_10%,0.10_	0.1010	0.2644	0.1185	0.1410	0.1435	0.1260	0.0900	0.2679	0.4254	0.7743
Entr-RB_15%,0.01_	0.1095	0.3064	0.1420	0.1685	0.1820	0.1785	0.1095	0.1915	0.6678	0.9873
Entr-RB_15%,0.05_	0.1095	0.3049	0.1405	0.1695	0.1805	0.1775	0.1095	0.1935	0.6668	0.9883
Entr-RB_15%,0.10_	0.1140	0.3134	0.1440	0.1750	0.1900	0.1900	0.1165	0.1720	0.7003	0.9453
$p_{0}$	0.4444	0.4631	0.0000	0.3703	0.6351	0.6351	0.4444	0.1852	0.3704	0.6351
$p_{+}$	0.5556	0.5369	1.0000	0.6297	0.3649	0.3649	0.5556	0.0000	0.2963	0.0000
$p_{-}$	0.0000	0.0000	0.0000	0.0000	0.0000	0.0000	0.0000	0.8148	0.3333	0.3649
	Equi-RB_5%,0.01_	Equi-RB_5%,0.05_	Equi-RB_5%,0.10_	Equi-RB_10%,0.01_	Equi-RB_10%,0.05_	Equi-RB_10%,0.10_	Equi-RB_15%,0.01_	Equi-RB_15%,0.05_	Equi-RB_15%,0.10_	
Bench_*MI*,5%_										
Bench_*MI*,10%_										
Bench_*MI*,15%_										
Equi-NoRB_5%_										
Equi-NoRB_10%_										
Equi-NoRB_15%_										
Entr-NoRB_5%_										
Entr-NoRB_10%_										
Entr-NoRB_15%_										
Equi-RB_5%,0.01_										
Equi-RB_5%,0.05_	0.2959									
Equi-RB_5%,0.10_	0.8273	0.6783								
Equi-RB_10%,0.01_	0.1015	0.1090	0.1100							
Equi-RB_10%,0.05_	0.0895	0.0975	0.0915	0.0250						
Equi-RB_10%,0.10_	0.1000	0.1090	0.1030	0.6078	0.4554					
Equi-RB_15%,0.01_	0.2299	0.2414	0.2354	0.8818	0.7778	0.8298				
Equi-RB_15%,0.05_	0.2259	0.2389	0.2334	0.8933	0.7848	0.8408	0.8873			
Equi-RB_15%,0.10_	0.2114	0.2284	0.2214	0.9563	0.8598	0.9183	0.1545	0.1685		
Entr-RB_5%,0.01_	$0.0260^{-}$	$0.0230^{-}$	$0.0250^{-}$	$0.0035^{-}$	$0.0035^{-}$	$0.0040^{-}$	$0.0145^{-}$	$0.0145^{-}$	$0.0140^{-}$	
Entr-RB_5%,0.05_	$0.0235^{-}$	$0.0220^{-}$	$0.0230^{-}$	$0.0035^{-}$	$0.0035^{-}$	$0.0035^{-}$	$0.0135^{-}$	$0.0140^{-}$	$0.0130^{-}$	
Entr-RB_5%,0.10_	$0.0230^{-}$	$0.0195^{-}$	$0.0225^{-}$	$0.0030^{-}$	$0.0030^{-}$	$0.0030^{-}$	$0.0125^{-}$	$0.0130^{-}$	$0.0130^{-}$	
Entr-RB_10%,0.01_	0.6448	0.6178	0.6413	$0.0345^{-}$	$0.0280^{-}$	$0.0315^{-}$	0.0525	0.0510	$0.0470^{-}$	
Entr-RB_10%,0.05_	0.6293	0.5979	0.6273	$0.0320^{-}$	$0.0260^{-}$	$0.0285^{-}$	$0.0490^{-}$	$0.0480^{-}$	$0.0450^{-}$	
Entr-RB_10%,0.10_	0.6343	0.6068	0.6298	$0.0325^{-}$	$0.0245^{-}$	$0.0280^{-}$	$0.0480^{-}$	$0.0475^{-}$	$0.0450^{-}$	
Entr-RB_15%,0.01_	0.9043	0.8698	0.8958	0.0865	0.0750	0.0800	0.1100	0.1085	0.1015	
Entr-RB_15%,0.05_	0.9033	0.8743	0.8978	0.0870	0.0735	0.0790	0.1080	0.1055	0.0995	
Entr-RB_15%,0.10_	0.9438	0.9153	0.9353	0.1020	0.0875	0.0960	0.1280	0.1265	0.1180	
$p_{0}$	0.5291	0.5291	0.5291	0.3704	0.2778	0.3704	0.4938	0.4938	0.2778	
$p_{+}$	0.0476	0.1217	0.0476	0.4444	0.5741	0.4815	0.4074	0.4074	0.5741	
$p_{-}$	0.4233	0.3492	0.4233	0.1852	0.1481	0.1481	0.0988	0.0988	0.1481	
	Entr-RB_5%,0.01_	Entr-RB_5%,0.05_	Entr-RB_5%,0.10_	Entr-RB_10%,0.01_	Entr-RB_10%,0.05_	Entr-RB_10%,0.10_	Entr-RB_15%,0.01_	Entr-RB_15%,0.05_	Entr-RB_15%,0.10_	
Bench_*MI*,5%_										
Bench_*MI*,10%_										
Bench_*MI*,15%_										
Equi-NoRB_5%_										
Equi-NoRB_10%_										
Equi-NoRB_15%_										
Entr-NoRB_5%_										
Entr-NoRB_10%_										
Entr-NoRB_15%_										
Equi-RB_5%,0.01_										
Equi-RB_5%,0.05_										
Equi-RB_5%,0.10_										
Equi-RB_10%,0.01_										
Equi-RB_10%,0.05_										
Equi-RB_10%,0.10_										
Equi-RB_15%,0.01_										
Equi-RB_15%,0.05_										
Equi-RB_15%,0.10_										
Entr-RB_5%,0.01_										
Entr-RB_5%,0.05_	0.3854									
Entr-RB_5%,0.10_	0.7443	0.7783								
Entr-RB_10%,0.01_	0.0710	0.0630	0.0635							
Entr-RB_10%,0.05_	0.0815	0.0675	0.0685	0.5289						
Entr-RB_10%,0.10_	0.0830	0.0700	0.0695	0.7128	0.9618					
Entr-RB_15%,0.01_	0.0670	0.0600	0.0580	0.4659	0.4424	0.4554				
Entr-RB_15%,0.05_	0.0660	0.0590	0.0585	0.4684	0.4444	0.4599	0.9243			
Entr-RB_15%,0.10_	0.0575	0.0530	0.0530	0.3994	0.3714	0.3824	0.0880	$0.0330^{+}$		
$p_{0}$	0.1481	0.1587	0.1587	0.2469	0.2469	0.4444	0.4762	0.4762	0.4233	
$p_{+}$	0.0148	0.0000	0.0000	0.0864	0.0494	0.0000	0.0688	0.0688	0.1640	
$p_{-}$	0.8370	0.8413	0.8413	0.6666	0.7037	0.5556	0.4550	0.4550	0.4127	
